# Supplementary material for: Model-free estimation of completeness, uncertainties, and outliers in atomistic machine learning using information theory
Source: Nat Commun. 2025 Apr 29;16:4014. doi: 10.1038/s41467-025-59232-0 (PMC12041501; doi:10.1038/s41467-025-59232-0)
Supplement: Supplementary file 1 — Supplementary Information [file 41467_2025_59232_MOESM1_ESM.pdf]

Supplementary Information for: Model-free estimation of completeness, uncertainties, and outliers in atomistic machine learning using information theory

Daniel Schwalbe-Koda [1,2‡], **Sebastien Hamel** [1], **Babak Sadigh** [1], **Fei Zhou** [1], **Vincenzo Lordi** [1§]

[1]Lawrence Livermore National Laboratory, Livermore, CA 94550, United States
[2]University of California, Los Angeles, Los Angeles, CA 90095, United States

A. Supplementary Text

A.1. Derivation of the descriptor

Consider a representation $f : \mathcal{S} \to \mathcal{X}$ that maps atomic environments S into features $\mathbf{X} \in \mathcal{X}$, with $\mathcal{X} \subset \mathbb{R}^N$, and denote $f(S_i) = \mathbf{X}_i$. The effectiveness of the function f is often computed according to the following properties:[1]

1. **Invariance**: the representation encodes all symmetries of the system, i.e. given a symmetry operation $T : \mathcal{S} \to \mathcal{S}$, $f(S) = f(T(S))$.
2. **Completeness**: if two descriptors are equal, $f(S_i) = f(S_j)$, then the originating structures are equal up to a symmetry operation, $S_i = T(S_j)$.
3. **Metric**: the function f induces a metric d in the descriptor space $\mathcal{X}$.
4. **Continuity**: arbitrarily small displacements of atoms in $\mathcal{S}$ ideally lead to arbitrarily small distances between features in $\mathcal{X}$.
5. **Speed**: the representation should be fast to compute.
6. **Invertibility**: given $\mathbf{X}_i = f(S_i)$, it is possible to reconstruct S_i up to a symmetry operation.

The field has many representations, several of which exhibit different properties. Here, we derive a representation satisfying several of the properties above. The representation is inspired in simple distances distributions, which have been proven to satisfy these properties[1] and have been used for other materials systems[2].

A.1.1. Radial terms

As a first order approximation, one can obtain an invertible mapping between the structures by taking the pairwise distances between atoms, then reconstructing them using the information from all atoms at once[1]. In particular, to make a fixed-length representation, one can take the distances towards the k-nearest neighbors of each atom as a representation,

$$r_{ij} = \left\| \mathbf{r}_i - \mathbf{r}_j \right\|, \tag{S1}$$

where

‡E-mail: dskoda@ucla.edu
§E-mail: lordi2@llnl.gov

$$r_{i1} \leq r_{i2} \leq \ldots \leq r_{ik}. \tag{S2}$$

As distances between atoms infinitely far apart should be negligible according to a metric that relates to machine learning potentials, we take the representation as being the inverse of distances,

$$X_{ij}^{(1)} = \frac{w(r_{ij})}{r_{ij}}, \tag{S3}$$

where $X_{i1} \geq \ldots \geq X_{ik}$ and w is a cutoff function given by

$$w(r) = \begin{cases} \left[1 - \left(\frac{r}{r_c}\right)^2\right]^2 & , r \leq r_c \\ 0 & , r > r_c \end{cases}, \tag{S4}$$

where r_c is a cutoff distance. The weight function was chosen to satisfy two criteria: (1) fast convergence of the descriptor; and (2) scaling of each component of $\mathbf{X}_1$ approaching $r^{-3/2}$ to resembles the relationship between entropy and distances in an ideal gas. Figure S1 shows how the combination of the weight function from Eq. (S4) and the inverse distance $1/r_{ij}$ approximates a dependence of $r_{ij}^{-3/2}$.

In principle, given a large number of neighbors, the unit cell parameters, and r_c, an input structure S may be reconstructed from $f(S) = \{\mathbf{X}^{(1)}\}$ up to an isometry.[1]

A.1.2. Cross terms

As the structure can only be reconstructed from the set of representations of all neighbors, increasing the amount of information in each local environment is desirable. This would also allow us to distinguish between environments containing the same set of nearest-neighbor distances, but different angles. One way to do so is to incorporate distances between atoms in a neighborhood of i,

$$X_{ijl}^{(2)} = \frac{\sqrt{w(r_{ij})w(r_{il})}}{r_{jl}}, \tag{S5}$$

which is performed for each neighbor l of atom j in the neighborhood of i. The weights $w(r_{ij})$ and $w(r_{il})$ ensure that cross distances r_{jl} are less important far away from the center of the neighborhood. The square root ensures that $\mathbf{X}_i^{(2)}$ has the same scaling and units as $\mathbf{X}_i^{(1)}$. The final representation on a per-neighbor basis is

$$\mathbf{X}_{ij}^{(2)} = \left(X_{ij1}^{(2)}, \ldots, X_{ijk}^{(2)}\right), \tag{S6}$$

with the constraint $X_{ij1}^{(2)} \geq \ldots \geq X_{ij(k-1)}^{(2)}$. Finally, the second-order representation term for each atom is given by

$$\mathbf{X}_i^{(2)} = \frac{1}{k} \sum_j \mathbf{X}_{ij}^{(2)}. \tag{S7}$$

Where the radial distances $\mathbf{X}_i^{(1)}$ already suffice for reconstruction when all i's are considered (along with unit cell parameters),[1] the pairwise cross distances $\mathbf{X}_i^{(2)}$ may help reconstructing environments only from the vector $\mathbf{X}_i = \left(\mathbf{X}_i^{(1)}, \mathbf{X}_i^{(2)}\right)$, even though reconstruction may not be guaranteed for this descriptor. If instead of an average in Eq. (S7) we concatenated all vectors $\mathbf{X}_{ij}^{(2)}$, then reconstruction could be guaranteed within the sphere limited by r_c. Continuity of this descriptor is only possible when this cutoff r_c is smaller than the distance of the central atom to the $k-$th nearest neighbor, as switching between neighbors would create a discontinuity for the aggregated contributions. Figure S2 illustrates the pairwise distances relevant for the construction of this descriptor.

A.2. Dataset entropy

According to information theory, the entropy $\mathcal{H}$ of a distribution $p(x)$ is defined as

$$\mathcal{H} = -\int p(x) \log p(x) dx, \tag{S8}$$

where $p(x)$ is the distribution of data points, log is the natural logarithm, and the value of entropy is integrated over the entire data space $x \in \mathcal{X}$. In our case, using this definition has two problems: (1) it assumes the knowledge of the prior distribution $p(x)$ over the data space; and (2) it requires the integration over the entire configuration space. In our context of atomistic simulations, obtaining both requires exhaustively sampling the potential energy surfaces (PESes), which is undesirable.

Recently, Perez *et al.* proposed the use of entropy-maximization schemes for automatic dataset generation for machine learning (ML) interatomic potentials (IPs)[3,4]. To bypass the problems above, the authors approximated the entropy using a classical non-parametric estimation from the literature[5]. Up to a constant, that estimate is given by

$$\mathcal{H}\left(\{\mathbf{X}\}\right)_{\text{Perez}} = \frac{1}{n} \sum_{i=1}^{n} \log \left(n \min_j \left\| \mathbf{X}_i - \mathbf{X}_j \right\| \right), \tag{S9}$$

with $\mathbf{X}_i$ the descriptor of atom i, n the number of descriptors in the set $\{\mathbf{X}\}$, and $\|.\|$ the L_2 norm. This definition is similar to that from Oganov and Valle,[6]

$$\mathcal{H}\left(\{\mathbf{X}\}\right)_{\text{Oganov}} = \left\langle \log \left[1 - d\left(\mathbf{X}_i, \mathbf{X}_j\right) \right] \right\rangle, \tag{S10}$$

where d is a custom distance function between $\mathbf{X}_i, \mathbf{X}_j$, which assumes values between 0 and 1, and $\langle . \rangle$ is the average over all structures. One problem with these descriptions is that the nearest-neighbor distance in the descriptor space may not be a good approximation of the distribution density $p(x)$ and strongly depends on the choice of descriptor. Furthermore, the information penalty for overlapping descriptors (i.e., $\left\| \mathbf{X}_i - \mathbf{X}_j \right\| \to 0$ in Eq. (S9)) is $\mathcal{H} \to -\infty$, which may be undesirable. Often, when sampling PESes, oversampling certain configurations is expected, which can pose a problem to a measure of entropy that drastically penalizes any overlap between two points. Finally, the value of entropy is unbounded, assuming any real value. This prevents concrete analogies between atomistic datasets and information theory.

To bypass these problems, we model the distribution of data points $p(x)$ using a kernel density estimation (KDE) and use to quantify the entropy of a dataset. This first estimate is obtained by using a normalized kernel $K_h(\mathbf{X}, \mathbf{X}_i)$ with bandwidth h and averaging over all data points in a dataset $\{\mathbf{X}_i\}$,

$$p(\mathbf{X}) = \frac{1}{n} \sum_{i=1}^{n} K_h(\mathbf{X}, \mathbf{X}_i). \tag{S11}$$

Then, as sampling the input space $\mathcal{X}$ is undesirable when calculating the integral in Eq. (S8), we propose an empirical estimate[5] given by

$$\mathcal{H}\left(\{\mathbf{X}\}\right) = -\frac{1}{n} \sum_{i=1}^{n} \log p(\mathbf{X}_i). \tag{S12}$$

This equation corresponds to the empirical entropy for a set of points $\mathbf{X}_i \in \{\mathbf{X}\}$. Now, using Eq. (S11) to compute $\log p(\mathbf{X}_i)$ further simplifies this equation to

$$\mathcal{H}\left(\{\mathbf{X}\}\right) = -\frac{1}{n} \sum_{i=1}^{n} \log \left[\frac{1}{n} \sum_{j=1}^{n} K_h(\mathbf{X}_i, \mathbf{X}_j) \right], \tag{S13}$$

To finally compute the entropy, a Gaussian kernel between descriptors can be used,

$$K_h(\mathbf{X}_i, \mathbf{X}_j) = \exp\left(\frac{-\left\|\mathbf{X}_i - \mathbf{X}_j\right\|^2}{2h^2} \right), \tag{S14}$$

where $\|.\|$ is the L_2 norm. Along with Equation (S13), the computation of the kernel allows us to measure the information entropy of a given atomistic dataset with a single parameter h.

A.3. Properties of the entropy

The main difference between Eq. (S13) and Eqs. (S9), (S10) lies on the fact that overlapping (or completely dissimilar) points in Eq. (S13) do not lead to $\mathcal{H} \to -\infty$, which is desirable when sampling potential energy surfaces. Moreover, appropriate choice of a kernel K_h abstracts away from descriptor distances and maps the entropy back to the space of probability distributions. As a consequence, our entropy defined by Eq. (S13) exhibits the following properties:

- **Bounds**: the normalization of the kernel, $0 \leq K_h(\mathbf{X}_i, \mathbf{X}_j) \leq 1$, implies that $1 \leq \sum_j K_h(\mathbf{X}_i, \mathbf{X}_j) \leq n$, so $\mathcal{H}$ is bounded between 0 and $\log n$.
- **Minimum entropy**: $\mathcal{H} = 0$ corresponds to a degenerate dataset created with multiple copies of a single $\mathbf{X}_i$, thus one that does not provide information about a space $\mathcal{X}$ but only for a single point. This is exactly what one expects from $p(x) \to \delta(x)$ in Eq. (S8).
- **Maximum entropy**: $\mathcal{H} = \log n$ corresponds to a dataset with zero overlap between data points, hence conveying maximal information. In information theory, this corresponds to distributions where all outcomes are equally likely.
- **Entropy grows with dataset size**: Because of the $\log n$ term, datasets composed by non-overlapping data points always bring more information as the training set grows.
- **Entropy can decrease as new points are added**: In addition to the $\log n$ effect, if new points overlap substantially with the existing dataset, the entropy of the new dataset may be smaller than the entropy of the original dataset.

- **Units**: because of the reliance on the probability distributions, the entropy has units (nats) and can be used to compare datasets and descriptors. For example, for the same datasets, incomplete descriptors should have lower entropy than complete ones, as the former map two points to the same representation. For the same descriptors, richer datasets should have higher entropy than redundant ones.

These entropy properties correspond exactly to those in the field of information theory and, as a consequence of Eq. (S8), also relate to some of those from statistical mechanics.

A.4. Differential entropy

In addition to the dataset entropy from Eq. (S13), one can compute the expected variation in entropy from adding a new point to the dataset even when the distances $\|\mathbf{X} - \mathbf{X}_i\|$ are not infinite. In information theory, this corresponds to how much information the new data brings to the dataset considering its current distribution of points. Considering an arbitrary point in Eq. (S13), we define the differential entropy $\delta\mathcal{H}$ of adding a point $\mathbf{Y}$ to a dataset $\{\mathbf{X}_i\}_{i=1,\ldots,n}$ as

$$\delta\mathcal{H}(\mathbf{Y}|\{\mathbf{X}_i\}) = -\log\left[\sum_{i=1}^{n} K_h(\mathbf{Y}, \mathbf{X}_i)\right]. \tag{S15}$$

This form is related to the functional derivative of the information entropy from Eq. (S8) with respect to the probability distribution $p(x)$,

$$\frac{\delta\mathcal{H}}{\delta p(x)} = -1 - \log p(x), \tag{S16}$$

thus representing the sensitivity of the entropy $\mathcal{H}$ with respect to variations of its distribution $p(x)$. In our work, we shift it for convenience by a constant $1 - \log n$ (partially due to the normalization of the kernel and $p(x)$) and adopt $\delta\mathcal{H} = \log n - \log p(x)$. Furthermore, the term "differential entropy" is usually employed in information theory to describe the entropy of a continuous probability distribution. In our case, we prefer to reserve this term to the quantity given by $\delta\mathcal{H}$ and avoid using different terms for continuous or discrete probability distributions.

Equation (S15) above has interesting properties for dataset analysis and construction:

- **There is no limit to "information novelty":** Contrary to $\mathcal{H}$ in Eq. (S13), $\delta\mathcal{H}$ does not have an upper bound. If the point $\mathbf{Y}$ has near-zero overlap with all points $\{\mathbf{X}_i\}$ of the existing dataset — and thus has maximal novelty — then $K_h(\mathbf{Y}, \mathbf{X}_i) \to 0$ and $\delta\mathcal{H} \to +\infty$.
- **Duplicating one isolated point from the training set brings zero information:** If a point $\mathbf{Y}$ overlaps perfectly with only one data point in $\{\mathbf{X}_i\}$, the sum over kernel values is one and $\delta\mathcal{H} = 0$.
- **Negative $\delta\mathcal{H}$ implies redundant information:** A point that overlaps with multiple points may have the summation over kernel values greater than one, leading to $\delta\mathcal{H} < 0$. The latter situation corresponds to points that are overrepresented in the dataset $\{\mathbf{X}_i\}$
- **Lower bound:** the differential entropy has a lower bound $-\log n \leq \delta\mathcal{H}$, where n is the size of the dataset $\{\mathbf{X}\}$. This can only be achieved in the case where $K_h(\mathbf{Y}, \mathbf{X}_i) = 1$, and represents the scenario where all points overlap. The result can be interpreted as an absolute threshold for dataset redundancy.

With the properties above, it follows that the differential entropy of the points in the training set is always smaller or equal to zero, which allows us to compute uncertainties without arbitrary thresholds.

The entropy of a system can be recovered from the values of $\delta\mathcal{H}$ by

$$\mathcal{H}(\{\mathbf{X}\}) = \log n - \frac{1}{n}\sum_{j=1}^{n} \delta\mathcal{H}(\mathbf{X}_j|\{\mathbf{X}\}). \tag{S17}$$

Importantly, however, the differential entropy $\delta\mathcal{H}$ cannot be used to measure the entropy $\mathcal{H}(\{\mathbf{X}_i\}_{i=1,\ldots,n+1})$ compared to $\mathcal{H}(\{\mathbf{X}_i\}_{i=1,\ldots,n})$ when the point $\mathbf{X}_{n+1}$ is added to $\{\mathbf{X}_i\}_{i=1,\ldots,n}$. As the new estimated probability distribution $p(x)$ changes given the knowledge of $\mathbf{X}_{n+1}$, the density $\frac{1}{n}\sum_j K_h(\mathbf{X}_i, \mathbf{X}_j)$ may change when the summation index is allowed to go from 1 to $n+1$ instead of 1 to n.

A.5. Entropy in the nearest-neighbors limit

In the limit of non-overlapping points, the sum over kernel values $K_h(\mathbf{X}_i, \mathbf{X}_j)$ from Eq. (S13) can be simplified to $K_h(\mathbf{X}_i, \mathbf{X}_i) = 1$ plus the nearest neighbor value,

$$\mathcal{H}(\{\mathbf{X}\}) \approx \log n - \frac{1}{n}\sum_{i=1}^{n} \log\left[1 + \max_{j\neq i} K_h(\mathbf{X}_i, \mathbf{X}_j)\right], \tag{S18}$$

thus resembling the result from Eq. (S9). The assumption of a nearest neighbor dominance expedites the calculation of the entropy. However, the result may not be accurate, as it requires points with small overlap in the descriptor space, an unusual assumption when dealing with PESes. On the other hand, computing all pairwise kernels $K_h(\mathbf{X}_i, \mathbf{X}_j)$ can be expensive for a large dataset $\{\mathbf{X}\}$. A good compromise is to implement the summation over the neighborhood $\mathcal{N}_k$ of $\mathbf{X}_i$, which contains the k-nearest neighbors of $\mathbf{X}_i$,

$$\mathcal{H}(\{\mathbf{X}\}) \approx -\frac{1}{n}\sum_{i=1}^{n} \log\left[\frac{1}{k}\sum_{\mathbf{X}_j\in\mathcal{N}_k(\mathbf{X}_i)} K_h(\mathbf{X}_i, \mathbf{X}_j)\right], \tag{S19}$$

and query the k-nearest neighbors with average complexity $\mathcal{O}(kd\log N)$, where N is the reference dataset size. Several approximations and nearest neighbors search methods can be employed to obtain the nearest neighbors in the feature space. In the results shown in Sec. A.12, we used an approach based on nearest neighbors graph, which can handle dataset sizes on the order of millions, and is helpful when performing uncertainty quantification (see Sec. B).

The use of approximate nearest neighbors for the computation of $\delta\mathcal{H}$ is analogous to that from $\mathcal{H}$,

$$\delta\mathcal{H}(\mathbf{Y}|\{\mathbf{X}\}) \approx -\log\left[\sum_{\mathbf{X}_j\in\mathcal{N}_k(\mathbf{Y})} K_h(\mathbf{Y}, \mathbf{X}_j)\right]. \tag{S20}$$

An immediate consequence of this approximation is that the value of $\delta\mathcal{H}$ is *overestimated*, as contributions from neighbors outside of the k-neighborhood of each vector are neglected. As the values of k increase, $\delta\mathcal{H}$ necessarily decreases, reaching a minimum when the full dataset size is used for its computation. Therefore, when used with the absolute threshold $\delta\mathcal{H} > 0$, the approximate $\delta\mathcal{H}$ are *conservative estimates* of the uncertainty. Some approximate nearest neighbor methods also have recall smaller than 100%, representing the case where some of the true nearest neighbors are not recalled

during the query. Nevertheless, less accurate $\delta\mathcal{H}$ are still overestimated with respect to an ideal nearest neighbor search. This demonstrates that, despite the approximations of truncating the expansion of $\delta\mathcal{H}$, this value can provide conservative estimates when used as an UQ metric.

A.6. Dependence of entropy with the bandwidth

The non-parametric estimation of the information entropy $\mathcal{H}$ described in Eq. (S13) requires fitting a KDE to the data distribution. In the current work, this selection is challenging due to two issues: (1) differences in density lead to changes in the metric space of the descriptors $\mathbf{X}$; and (2) differences in entropy can vary with the choice of bandwidth. To simplify the problem, we selected a bandwidth of 0.015 Å^{-1}, adopted as constant in this work (except in Sections A.11.2 and A.11.4). As described in the Methods, this corresponds roughly to the distance between two FCC environments ($k = 32$, $r_{\text{cut}} = 5$ Å) with an equilibrium lattice parameter of 3.58 Å and another with unit cell parameters rescaled by 1% (see Fig. S6). The use of this bandwidth to match different units of entropy is described in Sec. A.11.2.

A.7. Dataset diversity

As shown in Fig. 2 of the main paper, the dataset entropy depends on how frequent each environment is sampled in the configuration space. Therefore, entropy values can often reduce even as dataset sizes drastically increase. To create a measure of dataset *diversity* that relates to the support of the distribution rather than sampling frequency, we propose to express the diversity D as

$$D\left(\{\mathbf{X}\}\right) = \log\left[\sum_{i=1}^{n} \frac{1}{\sum_{j=1}^{n} K(\mathbf{X}_i, \mathbf{X}_j)}\right] = \log\left[\sum_{i=1}^{n} \exp\left(\delta\mathcal{H}_i\right)\right], \tag{S21}$$

where $\delta\mathcal{H}_i = \delta\mathcal{H}(\mathbf{X}_i|\{\mathbf{X}\})$. This analytical form is proposed to satisfy the following properties:

- **For non-overlapping datasets (i.e., uniform distributions), D recovers $\mathcal{H}$:** this can be demonstrated by verifying that, in datasets where $K(\mathbf{X}_i, \mathbf{X}_j) = \delta_{ij}$, $\delta\mathcal{H}_i = 0, \forall i$ and $D(\{\mathbf{X}\}) = H(\{\mathbf{X}\}) = \log n$.
- **An entirely new data point increases the summation in diversity by one:** this follows from the fact that, for a new point $\mathbf{X}_{(n+1)}$ that does not overlap with any of the other points $\mathbf{X}_i$, $\delta\mathcal{H}_{(n+1)} = 0$.
- **D has the same units of $\delta\mathcal{H}$,** which is determined by the base of the logarithm, and thus is nats for this work.
- **Repeating data points in the training set does not increase its diversity**, even if the entropy can be reduced. This follows from the summation of inverse of $p(\mathbf{X}_i)$, which approximately re-weights the distribution of data points based on their frequency according to other points.

Within this definition, the diversity D of a dataset represents the coverage of the configuration space. However, it does not express the same value as $\log n$, the maximum information entropy. Whereas $\log n$ is agnostic to the coverage of the space, D attempts to quantify exactly how many unique points are present in the system. For example, a degenerate system with $\mathcal{H} = 0$ also has $D = 0$ regardless of $\log n$.

A.8. Toy examples for QUESTS

A.8.1. 2D visualization of the entropy

To visualize the concepts of entropy and distributions, we sampled 100 points in a two-dimensional space from a 2D Gaussian with mean zero and covariance matrix equal to the identity. Then, we computed the values of $p(x)$ from a KDE and its corresponding $\delta\mathcal{H}$ for each point on the 2D grid. Figures S3 and S4 show how the entropy $\mathcal{H}$ and the differential entropy $\delta\mathcal{H}$ behave with different distributions, bandwidths, and rescaling. If the objective was to reproduce the original Gaussian, as in a standard KDE, the choice of higher bandwidths (Fig. S3c) better approximates the actual distribution. While this example is more difficult to visualize in a high-dimensional space of atomistic environments, the distribution plots illustrate the analogous result that would happen to them.

A.8.2. Visualization and distance for the atomistic representation

The representation proposed in this work was created on a per-environment basis, with radial distances and cross distances, as explained in Section 2.1 above and shown in Fig. S2. The representation can be visualized in a single plot and used to differentiate between standard crystal structures, such as BCC, FCC, and HCP (Fig. S5). This descriptor can also be used upon modification of the original structure, such as strain. In Fig. S6, an FCC structure is strained between -5% and 5%, and the representation is visualized according to the applied strain. Interestingly, the distance between the descriptors and the applied strain varies near-linearly within this range.

A.8.3. Information entropy upon denoising

To exemplify how the entropy $\mathcal{H}$ and the descriptors can be used to quantify information within atomistic systems, we analyzed trajectories with decreasing diversity of atomic environments from Hsu et al.[7] Because the deviations of the atoms from their ideal lattice sites were removed with a denoising method to enable phase classification, we expect the values of $\mathcal{H}$ to decrease accordingly. To validate this intuition for $\mathcal{H}$, we computed the information entropy of four denoised phases of copper, as shown in Fig S7. As vibrational motion is removed from the system, the values of $\mathcal{H}$ for the crystalline phases FCC, BCC, and HCP decrease until reaching zero.[7] On the other hand, the liquid phase cannot be fully denoised, and the residual disorder is manifested in a higher information entropy value. This example illustratse a connection between configurational degrees of freedom and information $\mathcal{H}$ of atomistic structures.

A.9. Extended discussion on learning curves of the rMD17 dataset

Figure 2a of the main text discusses the trends in learning curves for different molecules in the rMD17 dataset, and Fig. S10 shows the results of all molecules. Within the discussion, ethanol can be perceived as an outlier for this trend. Despite being much smaller than the other molecules, its information entropy takes a long time to reach a maximum compared to its counterparts, thus increasing its information gap (Fig. S11), which is unexpected at first. To explain this result, we notice that the distribution of energies for the rMD17 dataset varies according to the molecule (Fig. S13). Molecules such as ethanol and malonaldehyde, despite small, have broader distributions compared to their counterparts, which correlates positively with higher information gaps (Fig. S14a). Thus, if we assume that energy distributions correlate with the accessible phase space on a per-system basis, then

the information gap correctly captures this effect for the molecules, including ethanol, explaining this counterintuitive outlier.

A.10. Cluster size distribution and classical nucleation theory

Beyond the critical nuclei described in the main text, we verify that the distribution of cluster sizes in the melt can also be predicted using our approach. Figure S28 shows that, for all pre-nucleation snapshots, the cluster sizes follow approximately a power law. An analytical expression derived from the CNT (Sec. B), when fit to the data, also matches the data distribution, with a predicted surface energy of about 0.104 J/m^2. While this value underestimates the experimental range of 0.177–0.221 J/m^2 (described in the main text) it is still remarkably close to the overall data considering the approximations of the cluster definition, surface-to-volume ratios, and other factors not accounted for in our approach. Obtaining this fit allows us to estimate system properties relevant for CNT without relying on direct measurements of the surface areas or Gibbs energies, thus providing useful insights on the physical phenomenon and further demonstrating the usefulness of our method.

A.11. Qualitative parallels between information entropy and thermodynamics

A.11.1. Information entropy and heat capacity

One of the simplest toy models for entropy is the Debye's model, which considers atoms interacting via harmonic potentials as a model for phonons and heat capacity. To obtain classical MD trajectories that match the physics from the Debye model (and the zero-point energy in quantum harmonic oscillators), we used the quantum thermal bath (QTB) implemented in LAMMPS.[8] We simulated a $10 \times 10 \times 10$ box of particles with the FCC structure, unit cell parameter of 3.645 Å, and mass of 62.5 g/mol. The bond terms are determined by the spring constant $k = 1.0$ eV/Å^2 and an equilibrium distance of 2.5775 Å. Bonds are created for particles that are between 2.0 and 3.0 Å apart. Then, the simulation is performed using a QTB at constant temperature, varying from 10 to 1000 K, $f_{\max} = 120$ ps^{-1}, $N_f = 100$, constant volume. The simulation was performed with an equilibration run of 300 ps and a production run of 100 ps using a timestep of 2 fs. The results are shown in Fig. S8. Although the entropy was obtained with a constant, low value of bandwidth (0.015 Å^{-1}), the entropy of a fitted Debye model matches closely that from the extracted simulations. Importantly, the entropy does not approach zero at 0 K due to the residual motion from the simulations that mimic the behavior of the zero-point energy.

A.11.2. Calibrating the information entropy to configuration entropy differences with the bandwidth

Because lower densities (higher atomic volumes) lead to lower distances in the descriptor spaces, calibrating the information and thermodynamic entropies may be used with a variable bandwidth that decreases with increasing atomic volume,

$$h(V) = a \exp\left(-bV^2\right) + c, \tag{S22}$$

where a, b, and c are unknown parameters. To estimate these parameters in a self-consistent way, we first performed simulations for the copper Einstein crystal at the NVT ensemble using the spring constant of 34.148 eV/Å^2 and for volumes from 6 to 50 Å^3/atom. Though this method may vary slightly with the choice of spring constant and temperatures, we observed that the selected bandwidth was

transferable across many systems without refitting, as discussed in Sec. A.11.4. The `fix ti/spring` command in LAMMPS was used with a value of λ that ensures that only the harmonic oscillator is considered in the simulation. Then, for each volume, the entropy of the system is computed for a range of bandwidths, varying from 0.010 to 0.090 Å^{-1}. As the entropy of the Einstein crystal is independent of the volume, we estimate the values of bandwidth that would keep the entropy reasonably constant across the range of volumes. Figure S29 shows the results of this investigation, and the fitted bandwidth prediction that rescales the (arbitrary) information entropy to the thermodynamically relevant units k_B/atom. This approach provides a systematic rescaling of the bandwidths, but unfortunately does not guarantee that this scaling is universal. Though all examples demonstrated in Sections A.11.3 and A.11.4 use this same scaling of the bandwidth, extension of this analysis beyond the toy examples discussed here will be an object of future investigation.

A.11.3. Information entropy and Lindemann's melting criterion

The Lindemann melting criterion is a well-known estimate for the melting point of materials.[9] According to this estimate, melting often happens when the ratio between the root mean square displacement (RMSD) of atoms with respect to their ideal lattice positions and the ideal interatomic distances approaches a constant factor, often around 0.10 for several metals. To verify if our method could reproduce these results, we gradually added noise to the positions of prototypical FCC, BCC, and HCP crystal structures. To obtain a statistically meaningful result, we employed a $25 \times 25 \times 25$ supercell for each of the structures, thus creating structures with 15,625 atoms. Then, for each level of noise, we computed the RMSD with respect to the ideal lattice sites, and the entropy of the noisy configuration. When a bandwidth of 0.057 Å^{-1} is used for the chosen volumes (Fig. S29), the resulting entropy is shown in Fig. S9. The results show that the entropy increases rapidly with the RMSD, and reaches values around 0.2 to 0.5 k_B with a normalized RMSD between 0.1 and 0.125. As typical entropies of solids prior to melting are around this range of 0.2 to 0.5 k_B, considering the entropy of a liquid around 1.3 k_B and melting entropies between 0.8 and 1.1 k_B, this result reproduces the intuition behind Lindemann's melting rule based on the entropy values. While many other factors are responsible for melting and the Lindemann criterion is a rough approximation, this toy example shows that the addition of noise to the system leads to entropy values compatible with expected ranges.

A.11.4. Comparing information theory and configuration entropy in toy systems

Using the information entropy defined in Eq. (5) of the main text, we verified whether non-parametric descriptor distributions derived from atomistic simulations can be used to predict the configuration component of thermodynamic entropy differences, i.e., the entropy due to uncertainty in positions, but not momenta nor composition. As a reference, we compared our analysis to entropy differences obtained from thermodynamic integration (TI) at constant temperature and volume/pressure. In particular, we computed phase diagrams for two well-known systems using classical simulations: the BCC-FCC phase boundary of Cu under high pressures and temperatures ($180 \leq P \leq 280$ GPa, $3600 \leq T \leq 4800$ K), and the α to β phase transformation of tin around 286 K. As entropy differences in solid-solid phase transformations tend to be small, often smaller than one Boltzmann constant k_B, obtaining exact entropies is essential to produce accurate phase diagrams from simulations. We started by performing MD simulations of Cu at low atomic volumes (6.5–8.0 Å^3/atom) in the NVT ensemble using a classical IP based on the embedded atom method (EAM) from Mishin et al.[10] For each temperature, volume, and phase, we obtained the Helmholtz free energy F within the TI method

and calculated the entropy by taking the derivative of the free energy with respect to the temperature (see Sec. B). Then, we computed the reference entropy difference between the BCC and FCC phases at each volume and temperature. To compare our information theoretic method against these TI-derived entropies, we performed MD simulations at the same (V, T) pairs, but without the coupled Hamiltonian used for the reference free energy; instead, we use Eq. (5) to analyze the information entropy of the descriptor distributions. At a bandwidth of approximately 0.082 Å^{-1} (see Fig. S29), the differences of information entropy approximate well those obtained with TI, with a mean absolute error (MAE) of 0.003 k_B/atom (Fig. S30b). Systematic deviations from the TI entropies are found as the volume increases, which could be an artifact of the selected bandwidth or functional form of the descriptors. Nevertheless, despite the approximations from the descriptors and KDE, we successfully recovered not only trends in thermodynamic values, but also the exact values of entropy differences for the BCC and FCC Cu. Using the energy values from the same simulations, we compared the phase boundary from both methods by mapping the Helmholtz free energy space F(V, T) into a Gibbs G(P, T) phase diagram (Methods). The BCC-FCC phase boundaries for Cu within the ranges of 180–280 GPa and 3600–4800 K are similar in shape and values despite the impact of small entropy errors in phase boundary shifts (Fig. S30c). Nevertheless, the phase boundary computed with the EAM potential and our QUESTS method is close to a phase boundary from the literature,[11] which was obtained using density functional theory (DFT) calculations and the quasi-harmonic approximation. Although an ideal free energy method would recover the exact boundary obtained from the TI, this comparison suggests that our method is within reasonable deviation from the original results.

To demonstrate that entropy differences can be computed beyond constant volume assumptions, we analyzed the phase transformation between the α and β phases of tin using the modified EAM (MEAM) potential from Ravelo and Baskes.[12] In this transformation, the density undergoes a change of approximately 20% from α- to β-Sn. First, we obtain the free energies with TI by mapping from the NVT to NPT space to ensure the consistency of the calculation at different values of λ (see Methods). On the other hand, our QUESTS approach allows computing information entropies directly from NPT simulations for each phase. From these results, we compute the free energy differences at each (P, T) as $\Delta G = \Delta U - T\Delta S + P\Delta V$, where U and V are obtained from the average energies and volumes during the simulations. Figure S30d shows that the free energy differences between our method and TI at constant pressure of 0.6 GPa are in reasonable agreement. Small errors in entropy differences in our method lead to a larger derivative of the free energy curve and overestimate the transition temperature by about 10%. Across a range of pressures and temperatures, the agreement between our method and TI is shown on the phase diagram of Fig. S30e. Although differences in transition temperatures suggest that the accuracy of our method can be further improved, this surprising agreement between descriptor distributions, information entropy, and statistical mechanics can spark future investigations on their connection.

A.12. Approximate computation of entropy and nearest neighbors

At larger scales, one drawback of computing entropy values is the necessity of computing kernel matrices between each test point and the entire training set. As the number of test points n_Y and training examples n_X grow, the cost of computing such matrices increases with $\mathcal{O}(n_X n_Y)$. To verify if this is a problem in a large atomistic model, we approximate the values of $\delta\mathcal{H}$ by truncating the summation in Eq. (7) and using an approximate nearest neighbors approach (see Supplementary Text, Section A.5), which decreases the complexity to $\mathcal{O}(n_Y N \log n_X)$, with N the number of neighbors in the descriptor space. As computing $\delta\mathcal{H}$ for each point $\mathbf{Y}$ is an embarrassingly parallel task,

the search can be distributed over different processes or threads to expedite the computation of this differential entropy. Figure S32 shows the total query times for the 32.5M environments of tantalum relative to the SNAP training set (4224 environments) as a function of approximate nearest neighbors parameters and parallelized over 56 threads. As the index is constructed to increase the accuracy of the approach (higher values of m, see Sec. B), larger query times are obtained, with the slowest time obtained when an index with $m = 100$ is created and $k = 30$ neighbors are queried for each of the 32.5M test environments. In that case, the computation of $\delta\mathcal{H}$ used a wall time of 1000 seconds when parallelized on 56 threads on 56 Intel Xeon CLX-8276L CPUs from the Ruby supercomputer. On the other hand, the fastest set of parameters ($m = 5$, $k = 3$, 56 threads) spent 100 seconds in the same hardware. As a reference, computing the exact $\delta\mathcal{H}$ values for the 32.5M atom system with respect to the SNAP dataset (4224 environments) takes a walltime of about 255 seconds using the same hardware and parallelization settings. While the approximate $\delta\mathcal{H}$ has better scaling for larger reference datasets and is not critical for the SNAP dataset, performing the nearest neighbor search adds additional time constants compared to the brute-force exact calculation of the true $\delta\mathcal{H}$. While the timings can further be improved with additional parallelization, code optimization, or use of GPU architectures, our results already demonstrate that the computation of the differential entropy, either in approximate or complete way, is accessible even for systems with a large number of environments. The results also illustrate the theoretical understanding (Sec. A.5) that the approximate values of $\delta\mathcal{H}$ are overestimated compared to the actual $\delta\mathcal{H}$ values, as shown in Fig. S33.

B. Supplementary Methods

These Supplementary Methods describe in more detail the additional results and calculations in the Supplementary Text.

Molecular dynamics simulations

All MD simulations were performed using the Large-scale Atomic/Molecular Massively Parallel Simulator (LAMMPS) software[13] (v. 2/Aug./2023). All simulations were performed using a 1 fs time step, except when stated otherwise.

Thermodynamic Integration: free energies of solids were computed by assuming a potential energy U_λ that couples a reference system with potential energy U_{ref} and the interacting one U_{IS} such that

$$U_\lambda = \lambda^2 U_{\text{IS}} + (1 - \lambda^2) U_{\text{ref}},$$

where the quadratic term λ^2 reduces the impact of sampling the space of (N, V, T, λ) with a uniform grid in λ, and thus creates a denser sampling around $\lambda = 0$ or $\lambda = 1$ which mitigates numerical integration errors. The Helmholtz free energy F of the interacting system is obtained first taking the derivative of the free energy of the system corresponding to U_λ with respect to λ,

$$\left(\frac{dF_\lambda}{d\lambda} \right)_{N,V,T} = \left\langle \frac{\partial U_\lambda}{\partial \lambda} \right\rangle_\lambda,$$

where U is the energy of the system. Integrating the expression above in λ, we obtain

$$F_{\text{IS}} = F_{\text{ref}} + \int_0^1 2\lambda \left\langle U_{\text{IS}} - U_{\text{ref}} \right\rangle_\lambda d\lambda,$$

where F_{ref} is known for any given temperature and volume. We adopted the Einstein crystal as the reference, and modified the `fix ti/spring`[14] in LAMMPS to obtain energies for each (V, T, λ) without using a switching function. Using this, we performed different simulations for each point of the grid, thus ensuring stricter convergence of the average energy differences $U_{\text{IS}} - U_{\text{ref}}$ for each λ. We used a uniform grid with a spacing of 0.02 for λ, leading to 51 data points for each phase and (V, T). Numerical integration was performed using the function from the QUADPACK library[15] interfaced by SciPy[16] (v. 1.11.1).

Entropy from TI: given the free energy computed using the TI method, the entropy by taking the derivative of the Helmholtz free energy with respect to the temperature,

$$S = -\left(\frac{\partial F}{\partial T}\right)_{N,V}.$$

As the free energy is not computed for an infinitely dense grid of (V, T) values, numerical derivatives can lead to inaccurate values of entropy. To mitigate this problem, we fit a quadratic 2D polynomial to the free energies as a function of the independent variables (V, T). The fit is performed using the Lasso method (L_1 regularization) for all polynomial features up to degree 2 using the scikit-learn[17] (v. 1.3.0) library, with $\alpha = 10^{-4}$ and a maximum of 10^6 iterations. Then, with the interpolated values of free energy, we obtain the entropy by taking the numerical derivatives of F with a fine grid of temperatures at each value of volume.

Phase diagrams from TI: given the convenience of using the NVT ensemble when performing thermodynamic integration calculations, we constructed P-T phase diagrams by first obtaining free energies in the (N, V, T) space. Then, using the value of average pressure for each volume, we map each point (P, T) into a volume V, and the resulting (V, T) into a free energy F. With these variables, we compute the Gibbs free energy as $G(P, T) = F(V(P, T), T) + P \times V(P, T)$. The functions $(V, T) \to F$ and $(P, T) \to V$ are performed as described before, thus using a two-dimensional polynomial regressor with degree 2 and L_1 regularization. We observed that direct mappings $(P, T) \to F$ led to numerical inconsistencies that drastically affected the outcomes of the phase diagram, especially given the small entropy differences between the phases. On the other hand, the step-wise mapping was found to be more numerically stable.

FCC-BCC Cu phase transition at high pressure: the phase boundary between the FCC and BCC phases of copper was simulated using the EAM potential from Mishin *et al.*[10] The phases were simulated at four volumes: 6.5, 7.0, 7.5, and 8.0 Å^3/atom, which correspond to the range of high pressures shown in Fig. S30b. All calculations were performed with $20 \times 20 \times 20$ supercells, leading to an FCC cell with 32,000 atoms and a BCC cell with 16,000 atoms. Simulations were performed at 9 temperatures between 3000 and 5000 K separated by 250 K, and 51 values of λ. The MD simulation was performed at the NVT ensemble with the Langevin thermostat implemented in LAMMPS[18] and a damping constant of 0.5 ps. The simulation was equilibrated for 100 ps before a 1 ns-long production run. During the production run, the pressure, energy, and the coupled energy $U_{\text{IC}} - U_{\text{ref}}$ were averaged for every time step, and later printed for post-processing in the TI approach. A spring constant of 34.148 eV/Å^2 was used to attach the Cu atoms to their ideal lattice sites, thus modeling the Einstein crystal.

MD trajectories for entropy calculations using our QUESTS method were performed in the NVT ensemble using the same temperatures and volumes as the TI method. Simulations used the same cell sizes as the TI, but had 100 ps-long production runs. Snapshots were saved every 2.5 ps. Entropy values were obtained by randomly sampling 200,000 environments of the saved trajectory with a variable bandwidth determined by their volume.

$\alpha-$ **to** $\beta-$**Sn phase transition:** the phase boundary between the α and β phases of tin was simulated using the MEAM potential from Ravelo and Baskes[12]. The equilibrium lattice parameters for these structures were found to be $a_\alpha = 6.483$ Å, $a_\beta = 5.830$ Å, and $c_\beta = 3.183$ Å. All calculations were performed with a $12 \times 12 \times 12$ supercell for α and $12 \times 12 \times 24$ for β, leading to a cell with 13,824 atoms each. For the TI, simulations were performed at three different volumes, corresponding to 98%, 100%, and 102% of the equilibrium volumes of each phase, 7 temperature values between 200 and 350 K spaced by 25 K, and 51 values of λ. The MD simulation was performed at the NVT ensemble with the Langevin thermostat implemented in LAMMPS[18] and a damping constant of 0.5 ps. The simulation was equilibrated for 40 ps before a 500 ps-long production run. During the production run, the pressure, energy, and the coupled energy $U_{\mathrm{IC}} - U_{\mathrm{ref}}$ was averaged for every time step, and later printed for post-processing in the TI approach. A spring constant of 2.0 eV/Å^2 was used to attach the Sn atoms to their ideal lattice sites, thus obtaining an ideal Einstein crystal as reference system.

Entropy calculations using our QUESTS method were performed in the NPT ensemble at 1 bar and same temperatures as the TI method. Simulations used the same cell sizes as the TI, but had 200 ps-long production runs, with snapshots saved every 10 ps. Entropy values were obtained by randomly sampling 100,000 environments of the saved trajectory with a constant bandwidth of 0.038 Å^{-1}, which corresponds to the bandwidth for the average of the volumes between the α and β phases (Fig. S29).

Classical nucleation theory analysis

Cluster size distribution: within the CNT, the expected number of clusters with radius r, denoted here as N_r, depends on the free energy difference between the solid and liquid phases ΔG_r,

$$N_r = N_0 \exp\left(-\frac{\Delta G_r}{\mathrm{k}_B T}\right),$$

with N_0 a constant, T the temperature, and k_B the Boltzmann constant. The free energy difference assumes spherical clusters and balances the volumetric free energy difference between the solid-liquid phases Δg_{SL} and the interfacial free energy γ_{SL},

$$\Delta G_r = \frac{4}{3}\pi r^3 \Delta g_{\mathrm{SL}} + 4\pi r^2 \gamma_{\mathrm{SL}}.$$

The fit in Fig. S28 is obtained by fitting the unknowns N_0, Δg_{SL}, and γ_{SL} for the equation

$$\log N_r = \log N_0 - \frac{4\pi r^3}{3\mathrm{k}_B T}\Delta g_{\mathrm{SL}} - \frac{4\pi r^2}{\mathrm{k}_B T}\gamma_{\mathrm{SL}}.$$

In this case, the values of r are estimated from the cluster size from the graph-theoretical approach and a density of 8960 kg/m^3. The fit was performed for the temperature of 917 K, which is approximately the temperature of solidification during the simulation, and used all cluster sizes of the first 120 steps of the simulation. The nucleation event is observed at the 125th step.

Uncertainty quantification

Conformal prediction: The prediction of errors from the values of $\delta\mathcal{H}$ described in Sec. 2.3 of the main text and shown in Fig. S19 can be performed using a conformal prediction.[42,43] The map between $\delta\mathcal{H}$ and the error was obtained by first randomly splitting each subset into two halves, then

performing a quantile regression[19] with the `statsmodels` package (v. 0.14.4) for one of the halves. We adopted the least absolute deviation method, which sets the quantile to 0.5 during this regression task. After that, we tested the model on the second half of the dataset, which was not used for the regression, and obtained the parity plots shown in Fig. S19.

Approximate nearest neighbors: The approximate nearest neighbors for feature vectors $\mathbf{X}$ demonstrated in Sec. A.12 was computed using PyNNDescent (v. 0.5.11), that implements a search strategy based on k-neighbor graph construction.[20] The number of neighbors used to construct the index is represented with m in Fig. S33. The default number of trees, leaf sizes, and other parameters were used in the construction of the index. Searches were performed using an epsilon value of 0.1.

C. Supplementary Figures

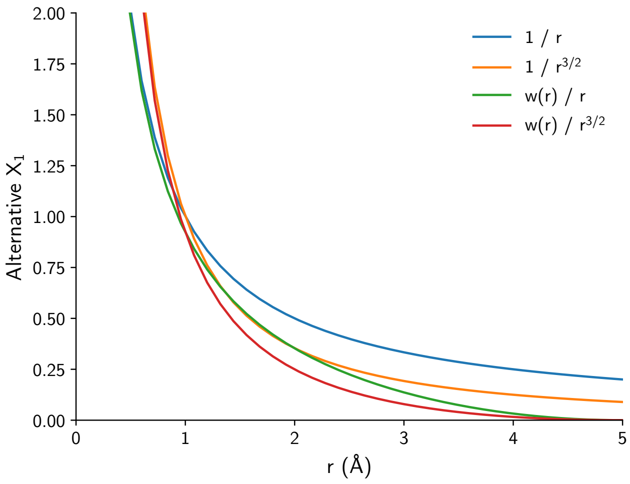

Fig. S1: Dependence of a proposed X_1 functional form according to interatomic distances r. A cutoff of 5 Å is used for the weight function $w(r)$.

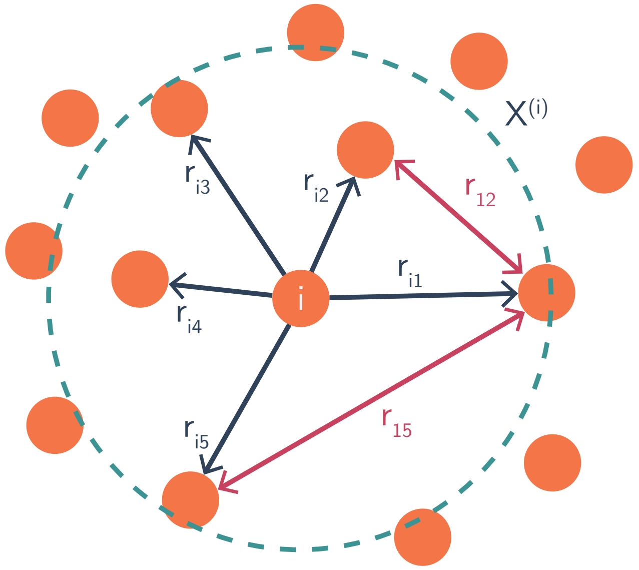

Fig. S2: Visualization of the distances r_{ij} used to create the $\mathbf{X}_1$ and $\mathbf{X}_2$ representation for atom i.

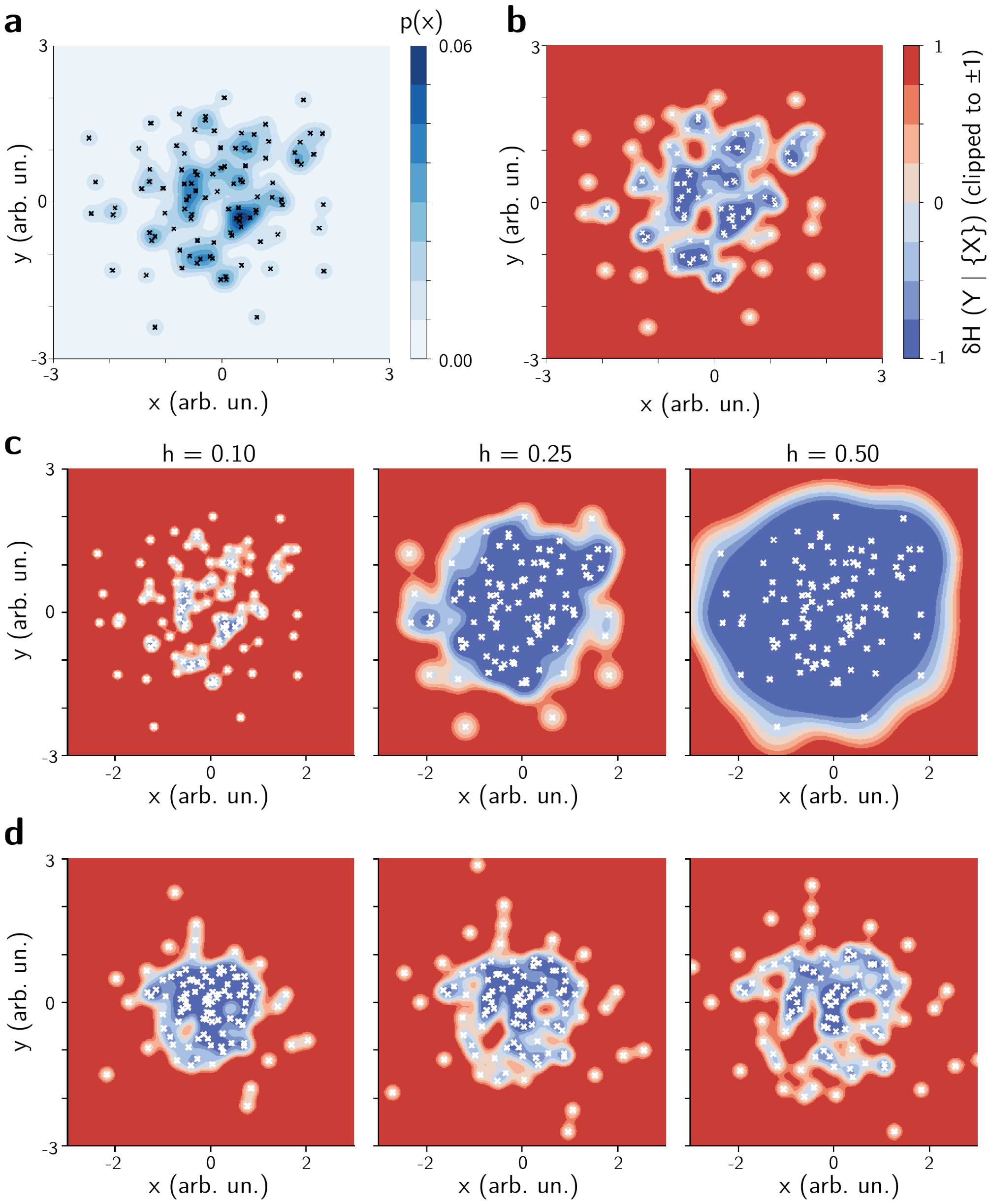

Fig. S3: 2D example of the kernel density estimate (KDE), bandwidth h, and entropy $\mathcal{H}$. **a**, estimated probability distribution $p(x)$ for a set of points (marked with crosses) in the 2D space (x, y positions of arbitrary units). **b**, the values of $p(x)$ can be mapped directly to the differential entropy $\delta\mathcal{H}$. This creates a common reference of $\delta\mathcal{H} > 0$ for points "outside" of the training set, shown here in red, and $\delta\mathcal{H} < 0$ for points "inside" the training set, shown in blue. **c**, effects of the bandwidth in estimating the probability distribution. A large bandwidth estimates the values as a single Gaussian, whereas a small bandwidth considers each point individually. **d**, effects of rescaling the coordinates of a distribution by a factor f in the entropy H. Denser distributions lead to lower entropy, whereas larger spread relates to higher entropy if the bandwidth is kept constant.

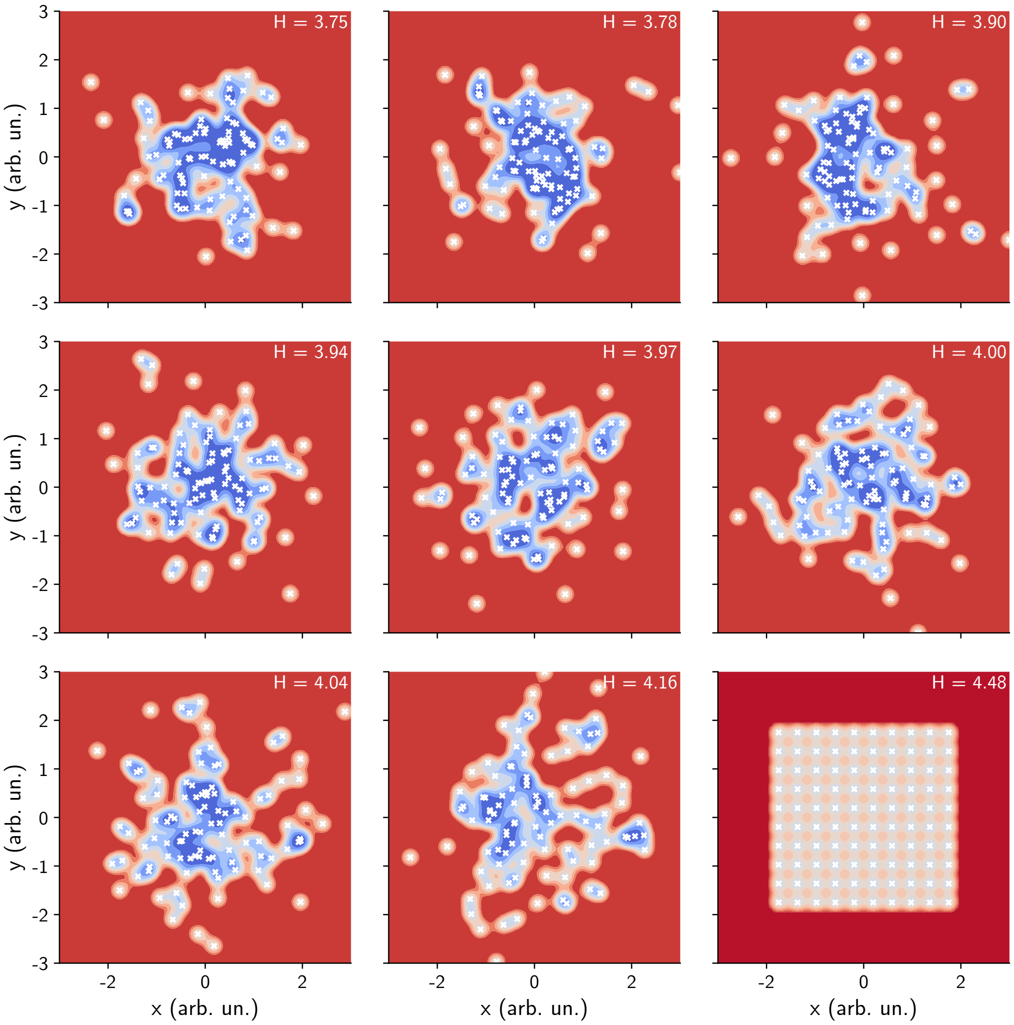

Fig. S4: 2D example of distributions with increasing entropy $\mathcal{H}$. The first eight distributions were generated randomly, then sorted according to their entropy. This provides a visual guide to interpreting values of lower entropy as more concentrated data points and higher entropy as larger spread. A regular occupation of the (2D) configuration space (bottom right) leads to the highest entropy among all examples. The color follows the same scale as Fig. S3b, with red points having differential entropy $\delta\mathcal{H} > 0$ and blue points having $\delta\mathcal{H} < 0$. The points are a toy example in the 2D space and thus have arbitrary units for the (x, y) coordinates.

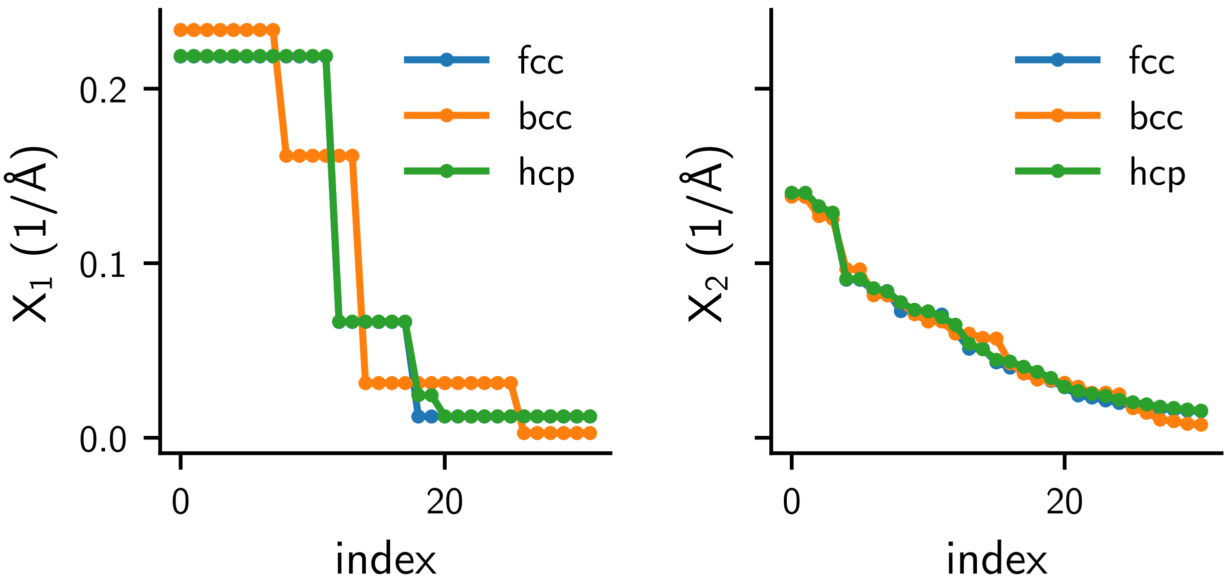

Fig. S5: Visualization of the $\mathbf{X}_1$ and $\mathbf{X}_2$ representation for face-centered cubic (FCC), body-centered cubic (BCC), and hexagonal close-packed (HCP) structures. The small differences between FCC and HCP can be seen only at neighbors further away from the origin.

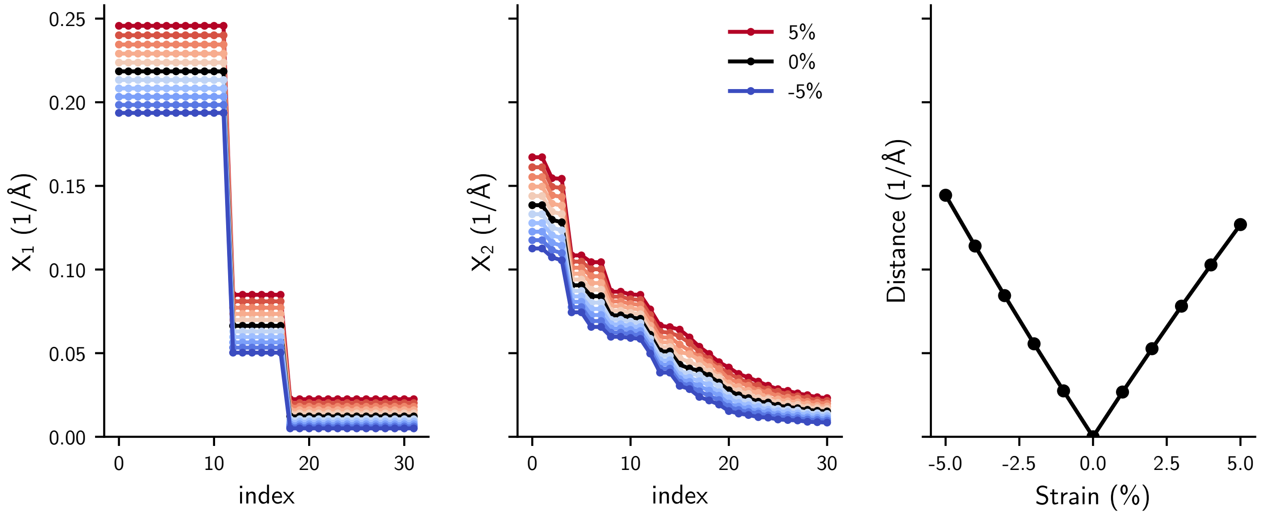

Fig. S6: Behavior of the $\mathbf{X}_1$ (left) and $\mathbf{X}_2$ (middle) representation for a face-centered cubic (FCC) structure under strain between -5% (expansion, blue) and 5% (compression, red). The Euclidean distance between the strained and reference structure is shown on the right. Within this range of uniform strains and this structure, the distance varies almost linearly.

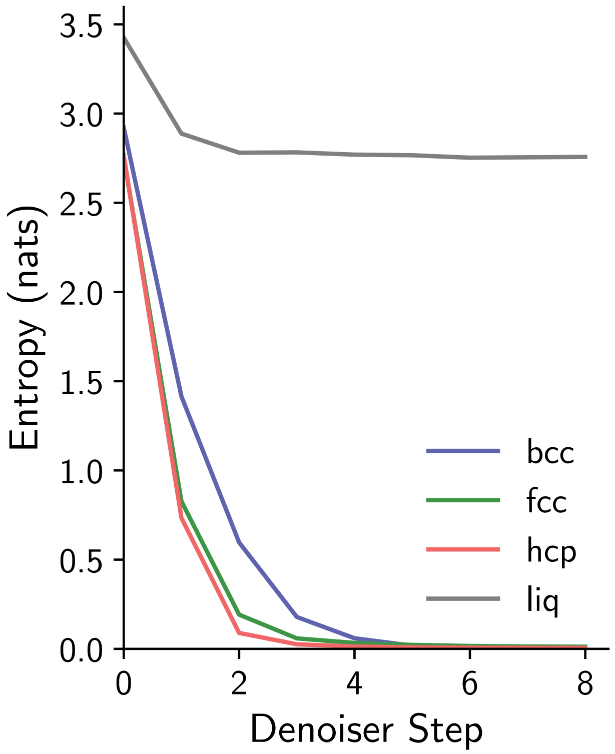

Fig. S7: Information entropy of four phases of copper — face-centered cubic (FCC), hexagonal close-packed (HCP), body-centered cubic (BCC), and liquid (liq) — for the denoised trajectories from Hsu *et al.*[7]

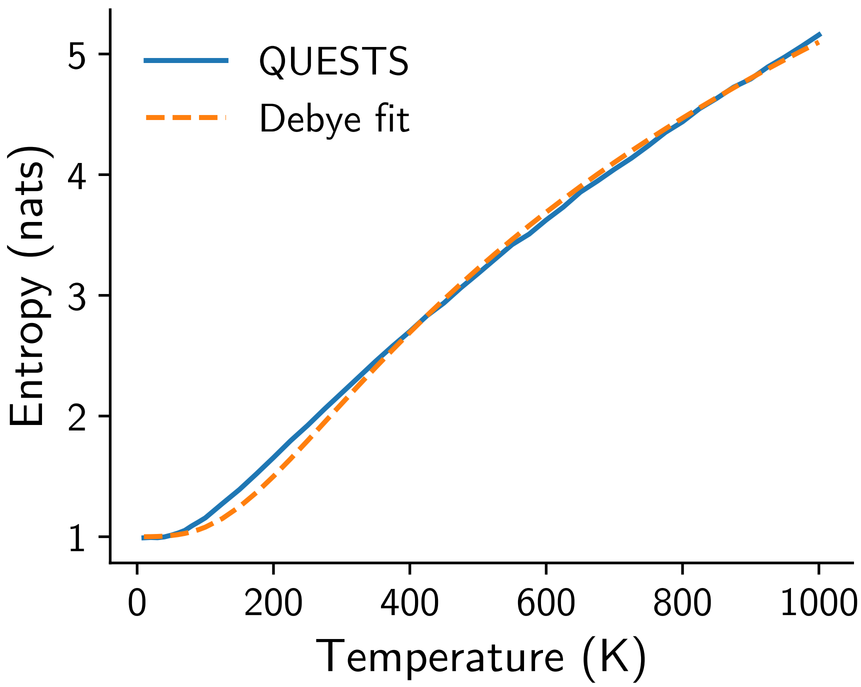

Fig. S8: Information entropy of particles interacting via harmonic bonds under a quantum thermal bath (QTB), as computed with our Quick Uncertainty and Entropy from STructural Similarity (QUESTS) method. At zero temperature, the entropy does not go to zero to simulate the effects of the zero-point energy. A fitted Debye model is shown with a dashed orange line.

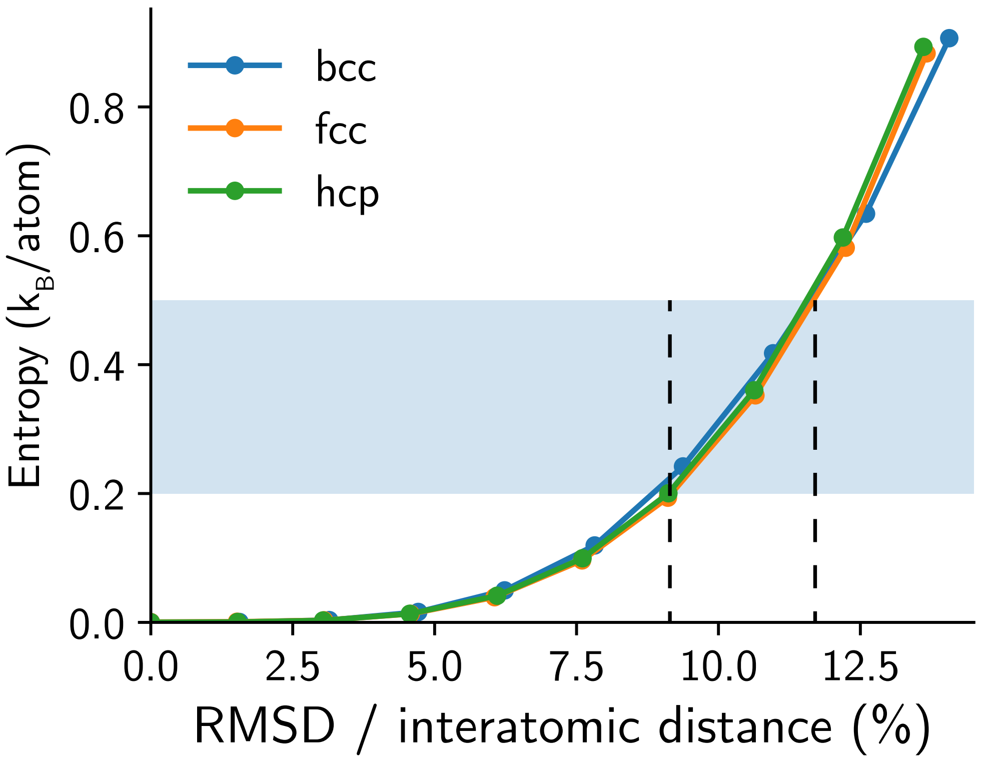

Fig. S9: Root mean square deviation (RMSD) of atoms in face-centered cubic (FCC), body-centered cubic (BCC), and hexagonal close-packed (HCP) structures, and their corresponding entropies calculated with our Quick Uncertainty and Entropy from STructural Similarity (QUESTS) method. The shaded area represents typical solid entropies prior to melting, and intersects the computed curves between 10–12.5% RMSD/interatomic distances, thus reproducing the Lindemann melting rule. The units of the entropy were obtained using a calibration of the bandwidth, as discussed in Section A.6.

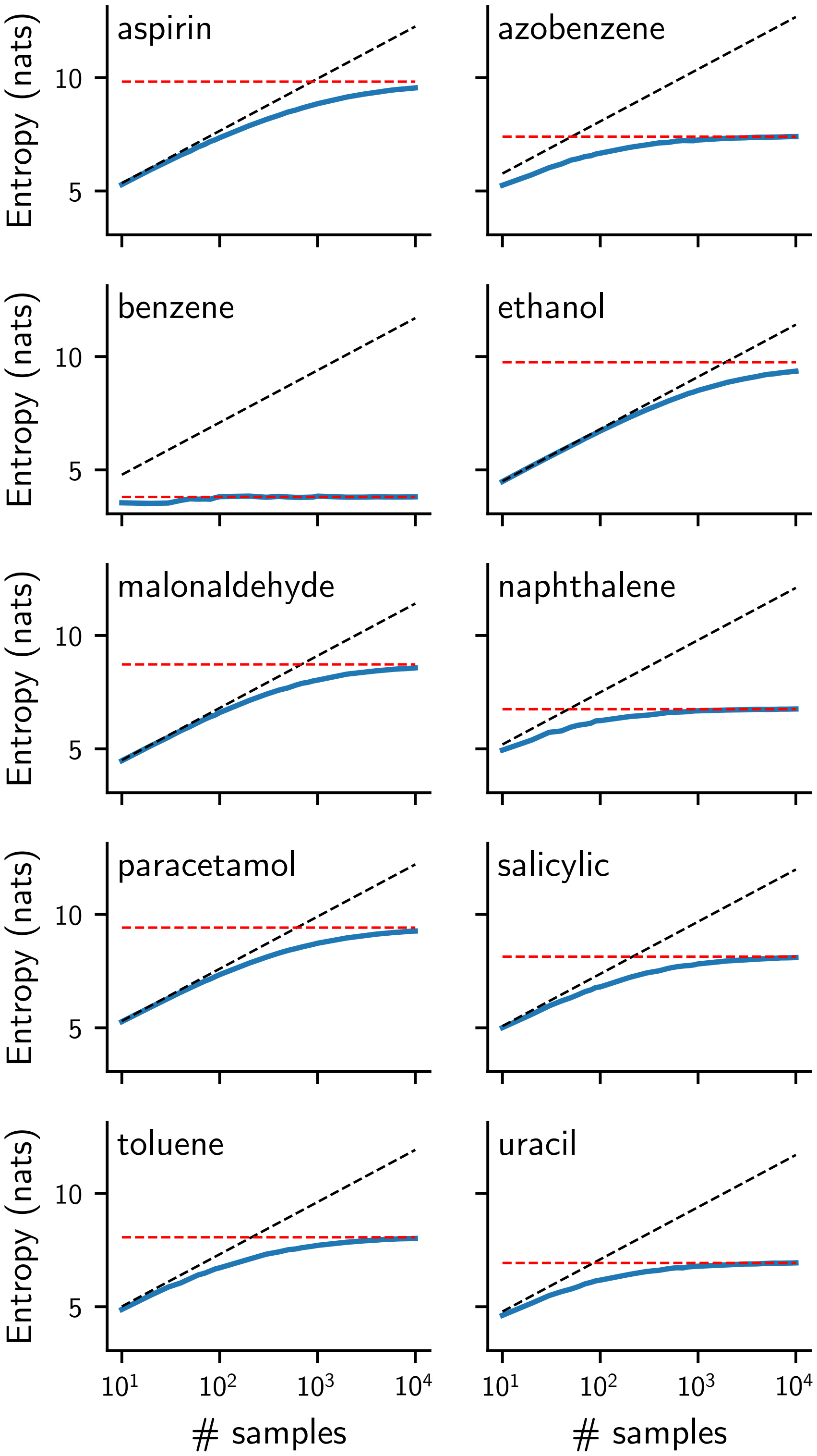

Fig. S10: Entropies for all molecules in the rMD17 dataset[21,22] as a function of training set size (# samples). The black dashed line is the behavior of $\log n$ considering the number of environments per molecule. The red line is the asymptote for the entropy.

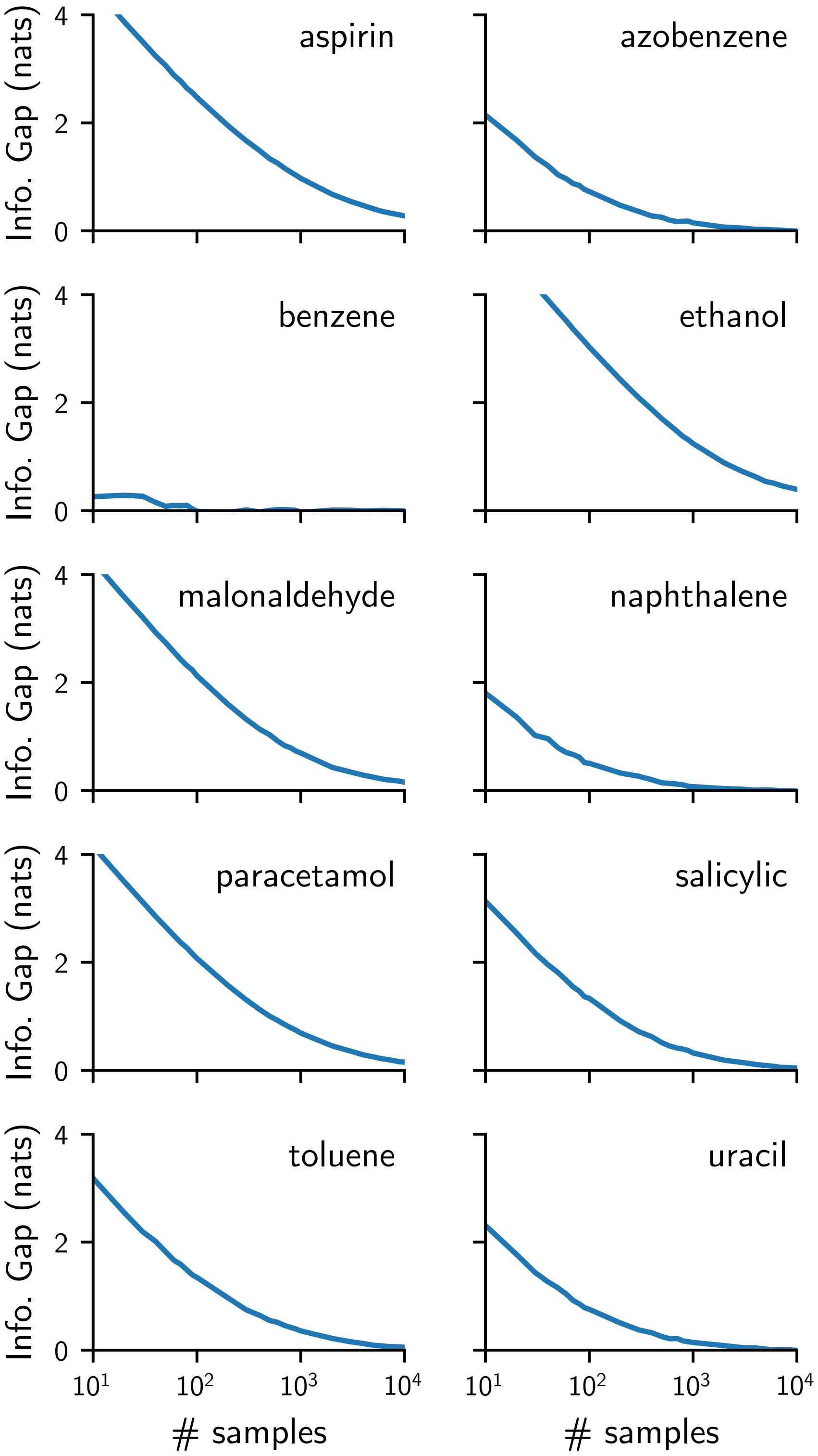

Fig. S11: Information gap for all molecules in the rMD17 dataset[21,22] as a function of training set size (# samples). The gap is defined as the asymptotic value of the information entropy minus the entropy value at a given number of samples. These curves show that, at a typical constant number of samples, the information gap varies substantially across molecules.

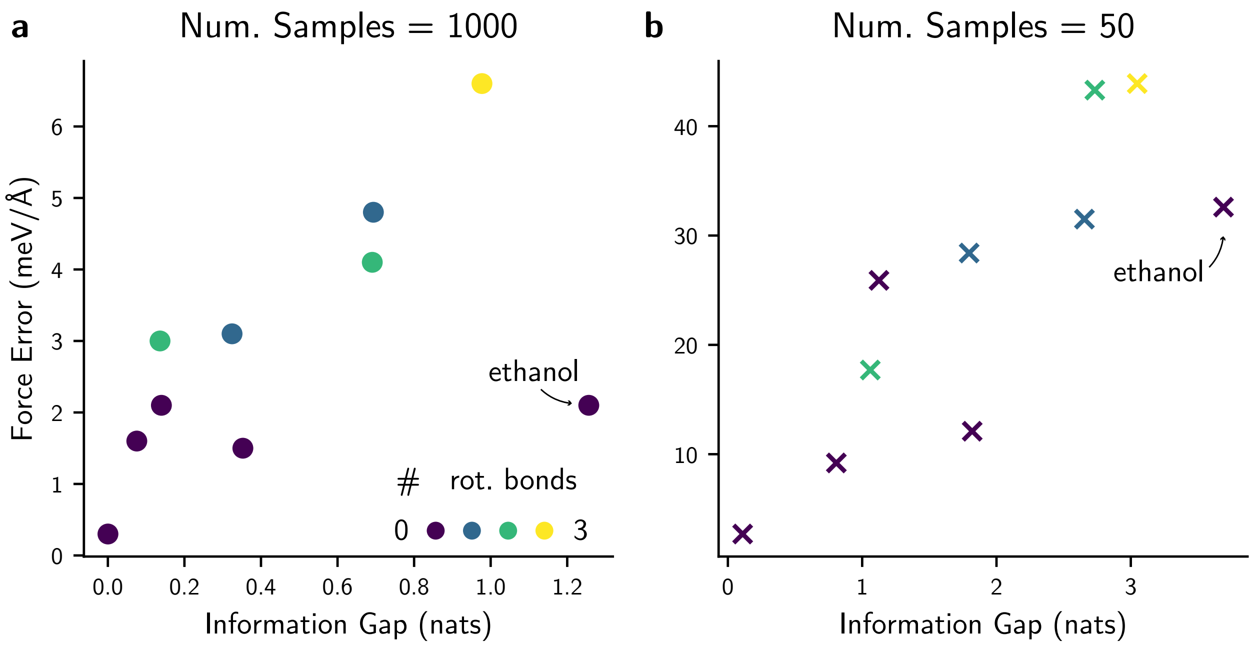

Fig. S12: Correlation between the force errors from a MACE model[23] and the information gap for each molecule in the rMD17 dataset.[21,22] The errors are shown separately for the model trained on two dataset sizes: **a**, 1000 samples and **b**, 50 samples. The color represents the number of rotatable bonds for each molecule. Ethanol is an outlier from the trend in **a**.

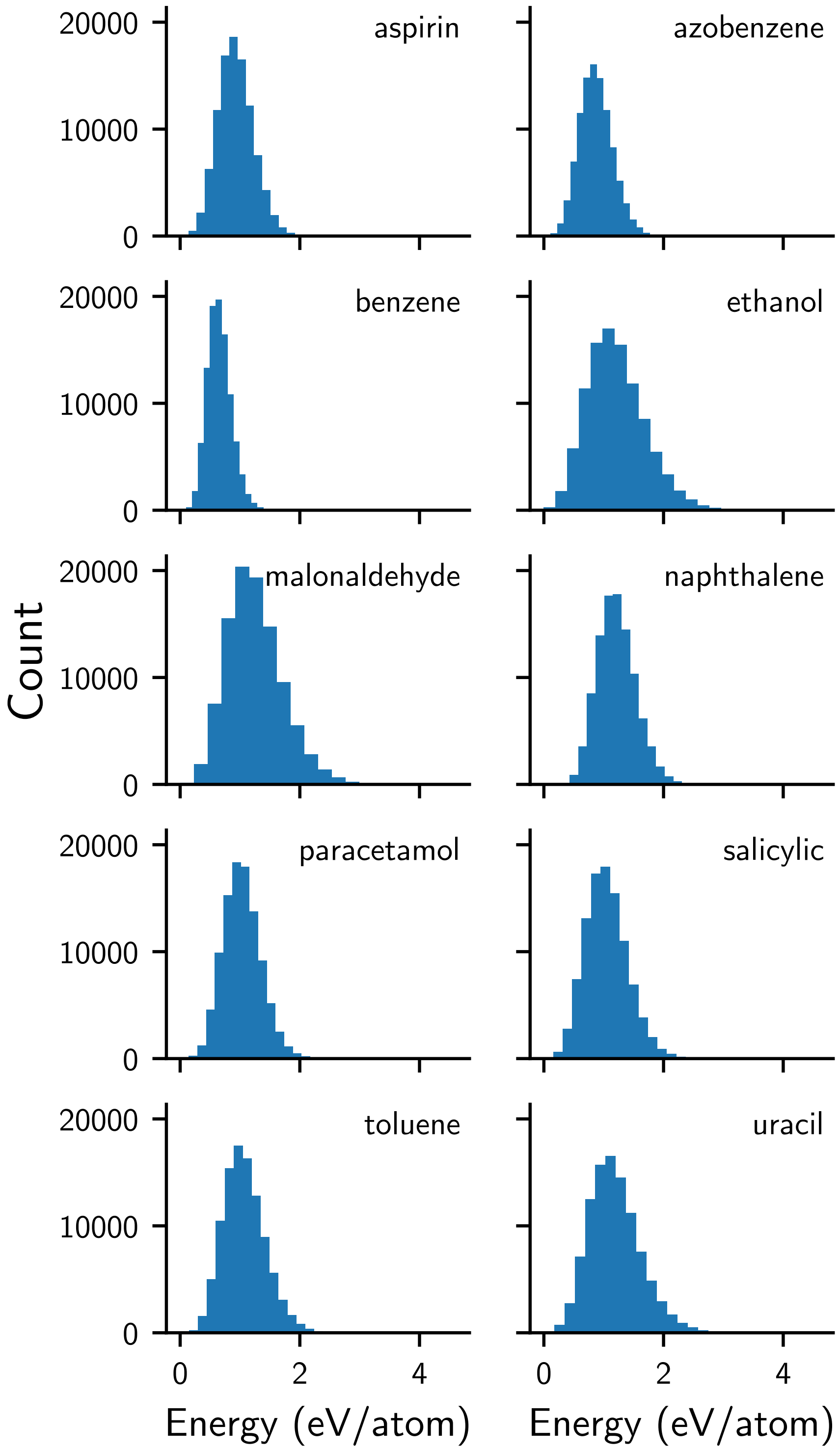

Fig. S13: Distribution of energies for the original rMD17 dataset.[21,22] Ethanol and malonaldehyde have larger standard deviations and longer tails towards higher energies.

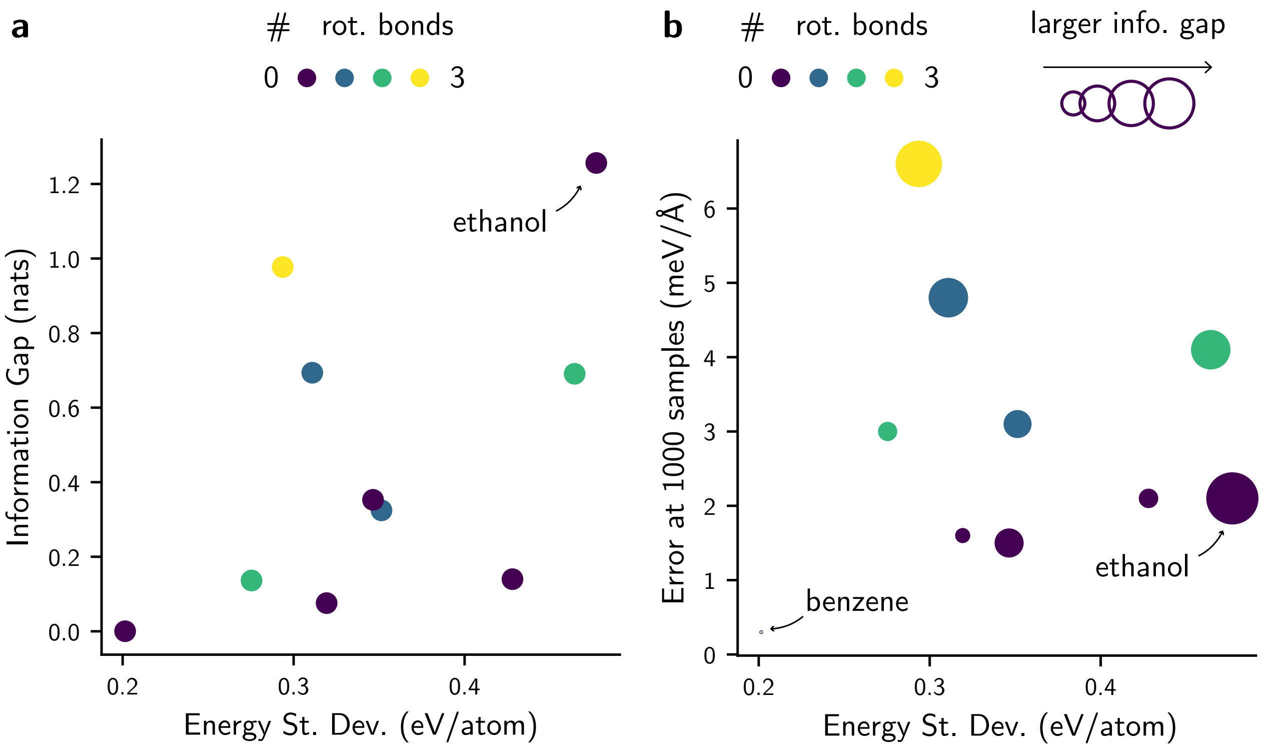

Fig. S14: **a**, Correlation between the information gap for each molecule in the rMD17 dataset[21,22] and the standard deviation of the distribution of energies in the dataset. **b**, Correlation between force errors from a MACE model,[23] the standard deviation of the distribution of energies in the dataset, and the information gap for each molecule in the rMD17 dataset[21,22] (represented with marker sizes). Brighter colors indicate more rotatable bonds. For the systems with zero rotatable bonds, the error is higher for wider distributions.

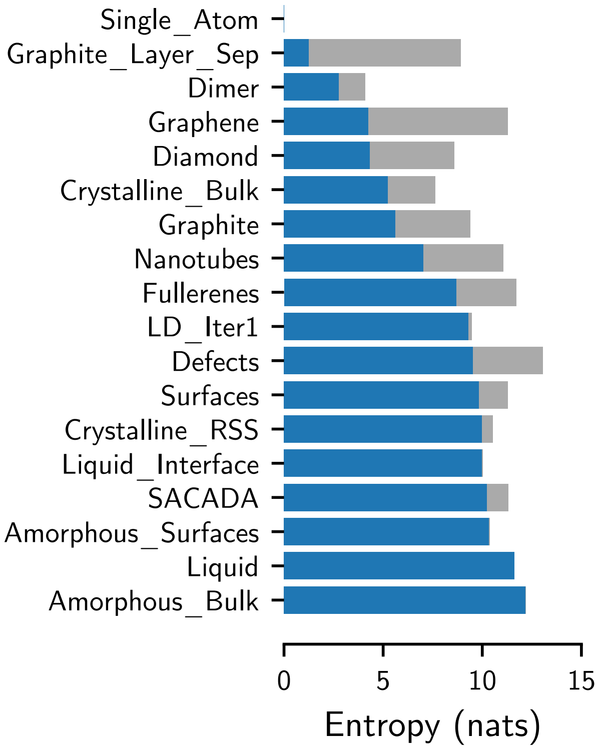

Fig. S15: Entropies for the subsets of the GAP-20 dataset for carbon[24] (blue) and maximum possible entropy for each subset ($\log n$, gray), where n is the number of environments in each subset. The entropy of the "Single_Atom" subset is zero, as it contains only a single environment.

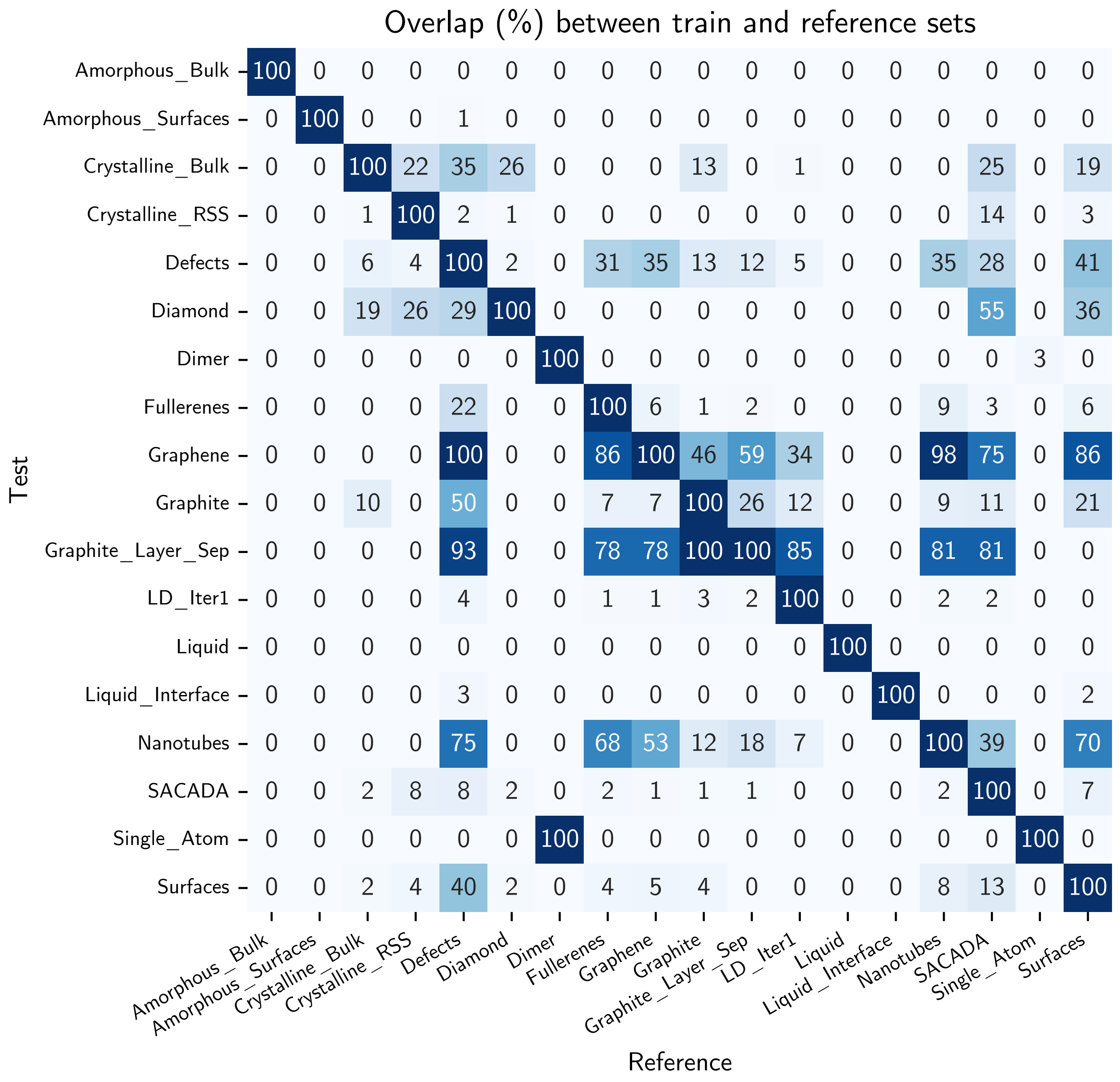

Fig. S16: Overlap between test and reference sets for the GAP-20 carbon dataset.[24]

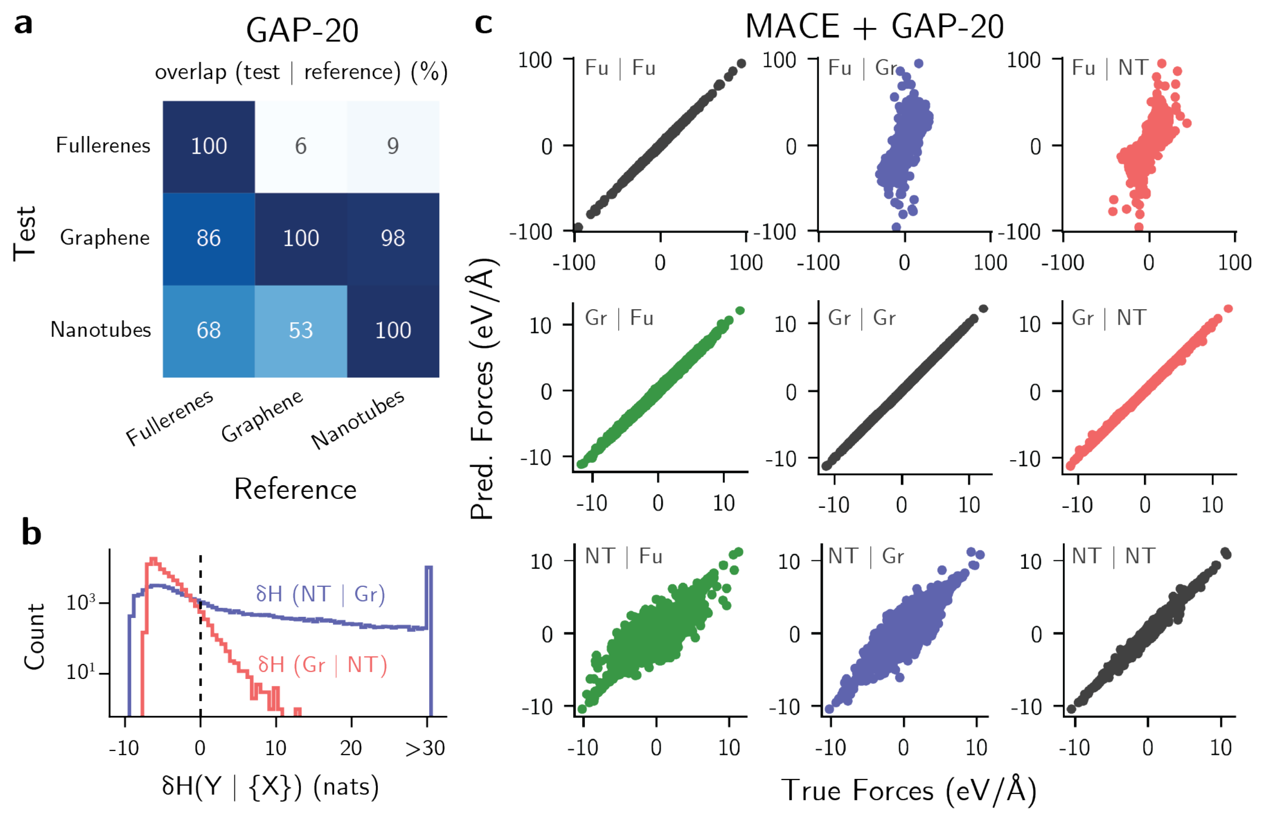

Fig. S17: **a**, Overlaps between different subsets of the GAP-20 dataset,[24] as also shown in Fig. 3a of the main text. The overlap is computed from the distribution of the differential entropy $\delta\mathcal{H}$ values such as the one in **b** that illustrates the asymmetry between the "Graphene" and "Nanotubes" datasets. **c**, parity plot illustrating the prediction errors of a MACE model trained on the "Fullerenes" (Fu), "Graphene" (Gr), and "Nanotubes" (NT) subsets, and tested on the others. The results reflect the trends of overlap in **a**.

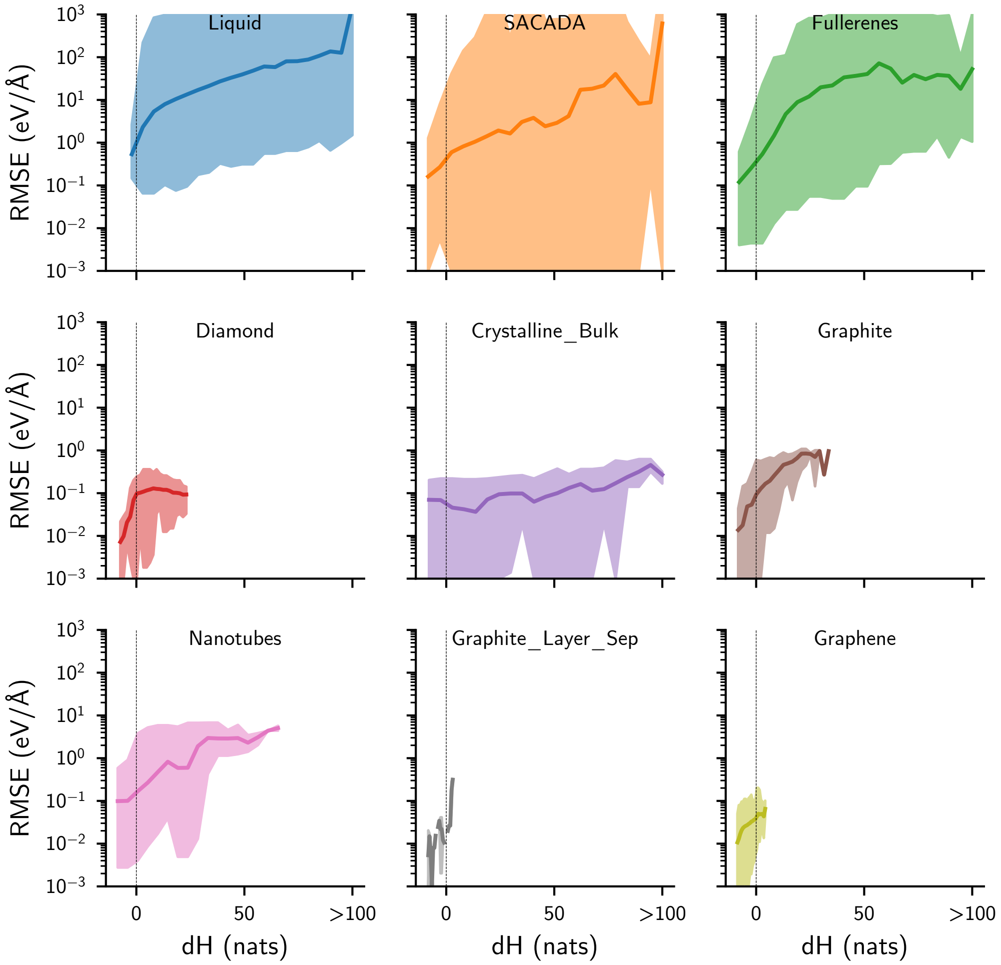

Fig. S18: Root-mean-square error (RMSE) of forces for MACE models[23] trained on the "Defects" GAP-20 dataset[24] and tested on other subsets. The test splits are sorted by increasing overlap with the training sets. The shaded area represents the range of the error distribution in each window of the differential entropy $\delta\mathcal{H}$. For clarity, small errors are truncated to be equal to 1 meV Å^{-1}, and the plot is truncated at a maximum error of 1000 eV Å^{-1}. Because some data points in the "Liquid" or "SACADA" subsets are infinitely far away from the "Defects" training set, their values of $\delta\mathcal{H}$ are also infinite. To avoid issues with the visualization, we clipped the values of $\delta\mathcal{H}$ at 100 for all sets.

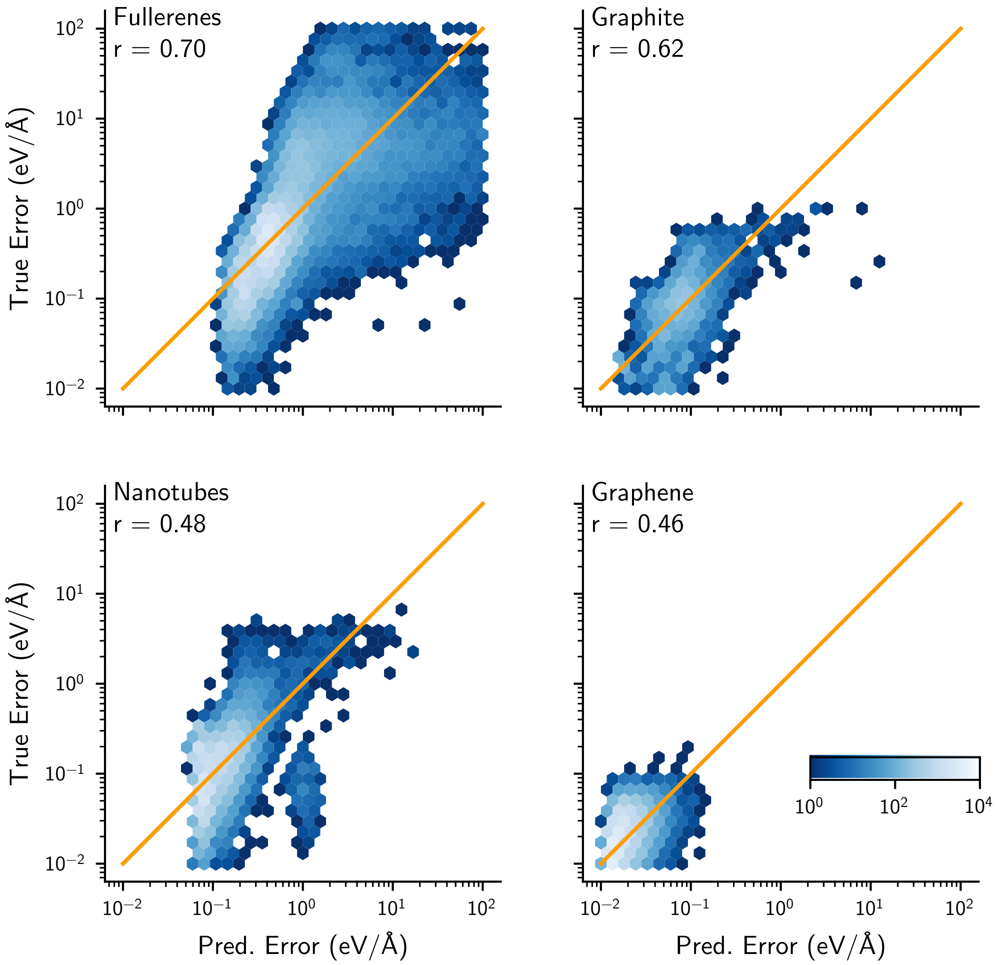

Fig. S19: Relationship between predicted root-mean-square error (RMSE) and actual RMSE of a MACE model[23] trained to the "Defects" subset of the GAP-20 dataset[24] and tested on other subsets. The predicted errors are obtained using a conformal prediction of the values of the differential entropy $\delta\mathcal{H}(\mathbf{X} \mid \text{Defects})$ for 50% of the dataset (randomly sampled), and tested for the other half of the dataset. The figure illustrates the results for the dataset split where the quantile regression was not fitted to. The values of r are the Pearson correlation coefficients for the log of the errors. The orange line showcases the perfect prediction $y = x$.

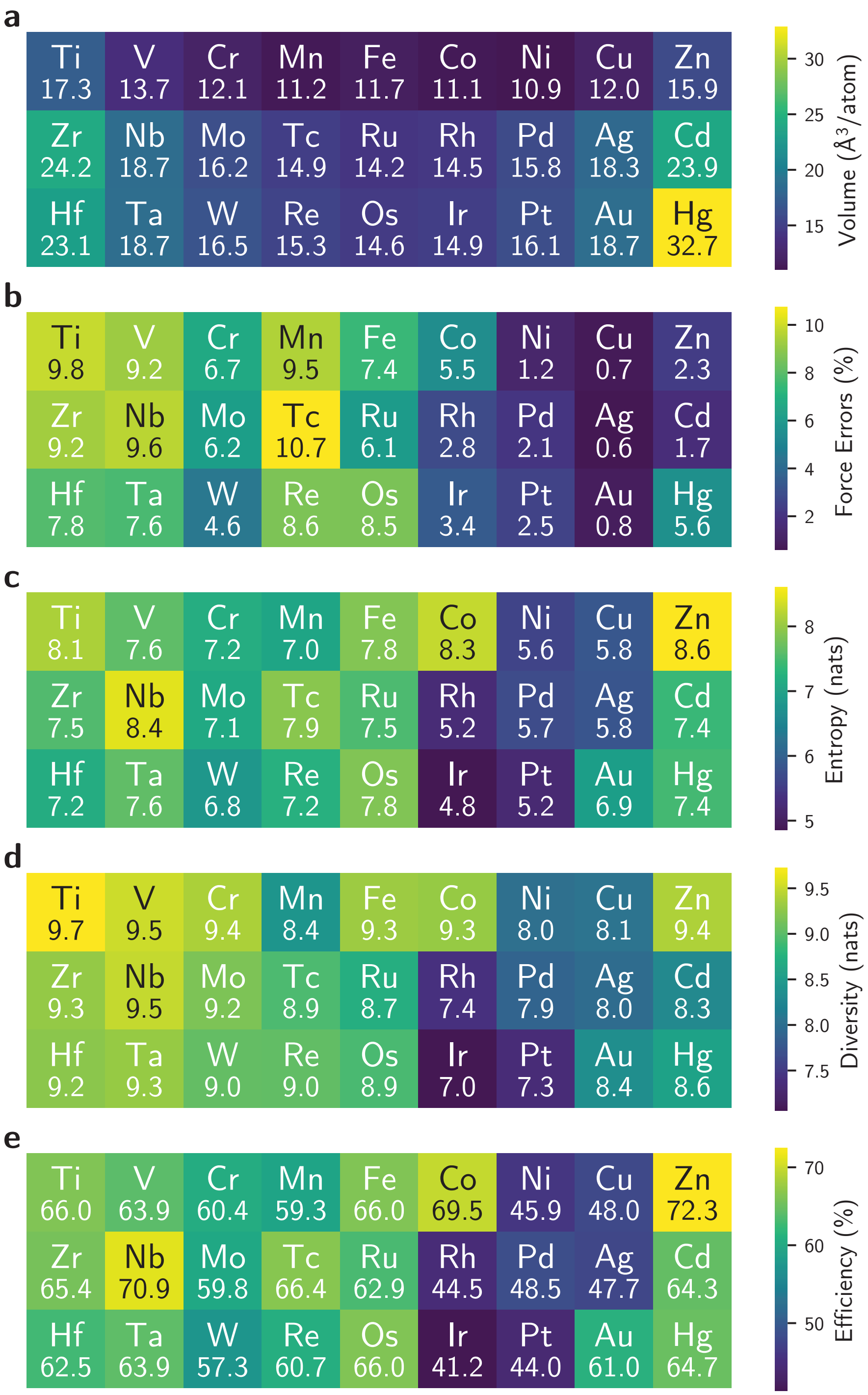

Fig. S20: Per-element values for the full TM23 dataset[25] (cold, warm, and melt subsets) for: **a**, atomic volume, **b**, relative force errors for the NequIP models[26] reported by Owen *et al.*,[25] **c**, entropy, **d**, diversity, and **e**, efficiency of each dataset. The efficiency is defined as the value of entropy divided by the maximum entropy of the dataset ($\log n$), where n is the number of environments in the dataset.

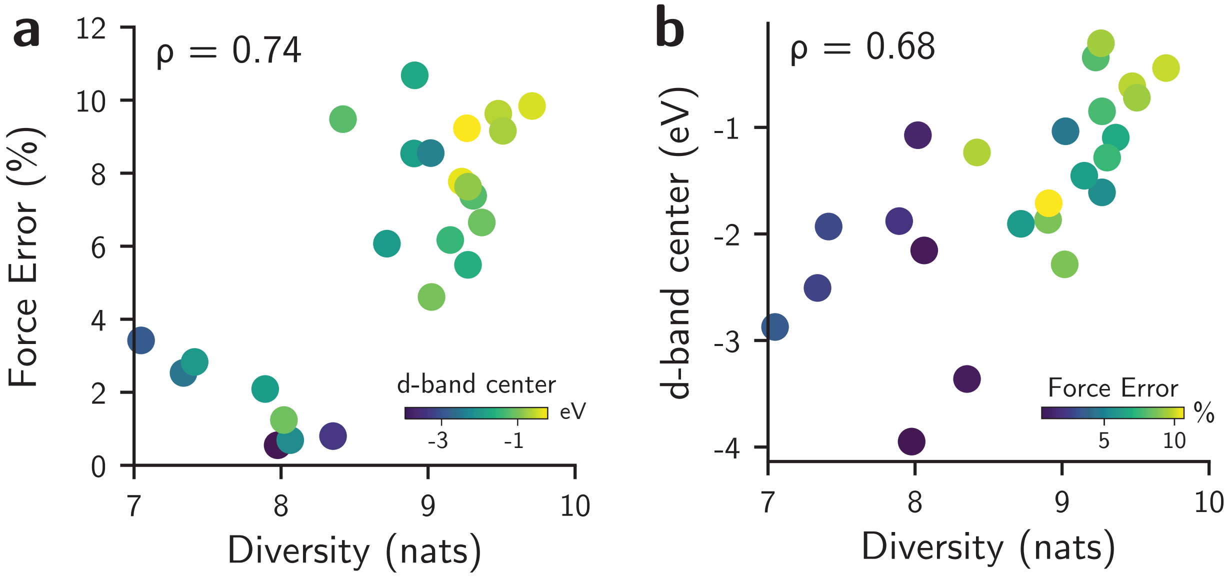

Fig. S21: Correlation between **a**, Force errors from Owen *et al.*[25] and diversity, and **b**, d-band center of the transition metals, as reported by Owen *et al.*,[25] and dataset diversity. The Pearson correlation coefficients ρ are also given. The results are described without group 12 elements, following the discussion by Owen *et al.*[25]

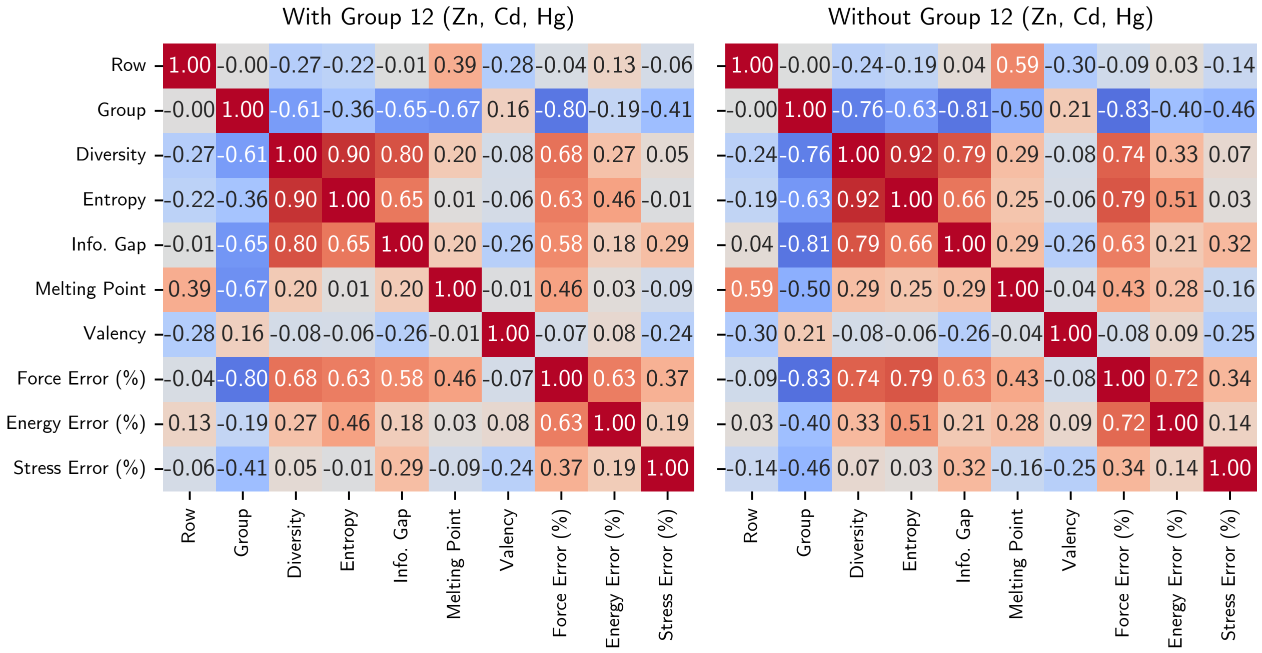

Fig. S22: Pearson correlation coefficients between quantities describing the TM23 dataset.[25] The results are described with (left) and without (right) group 12 elements, following the discussion by Owen *et al.*[25] In addition to the known trend between force errors and the group of the periodic table, we demonstrate that the diversity, entropy, and information gap also exhibit a reasonably strong correlation with the force errors.

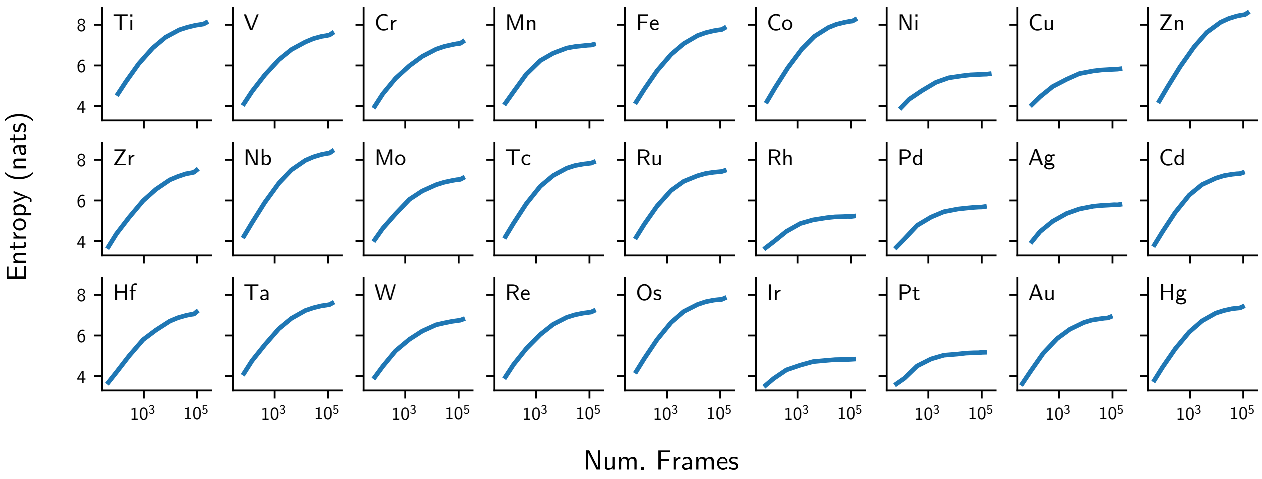

Fig. S23: Information (learning) curves for the TM23 dataset[25] obtained for the full subsets per element. The entropy is computed by averaging five different runs to obtain reliable statistics on each subset. Subsets are obtained by randomly sampling the number of frames from the main dataset.

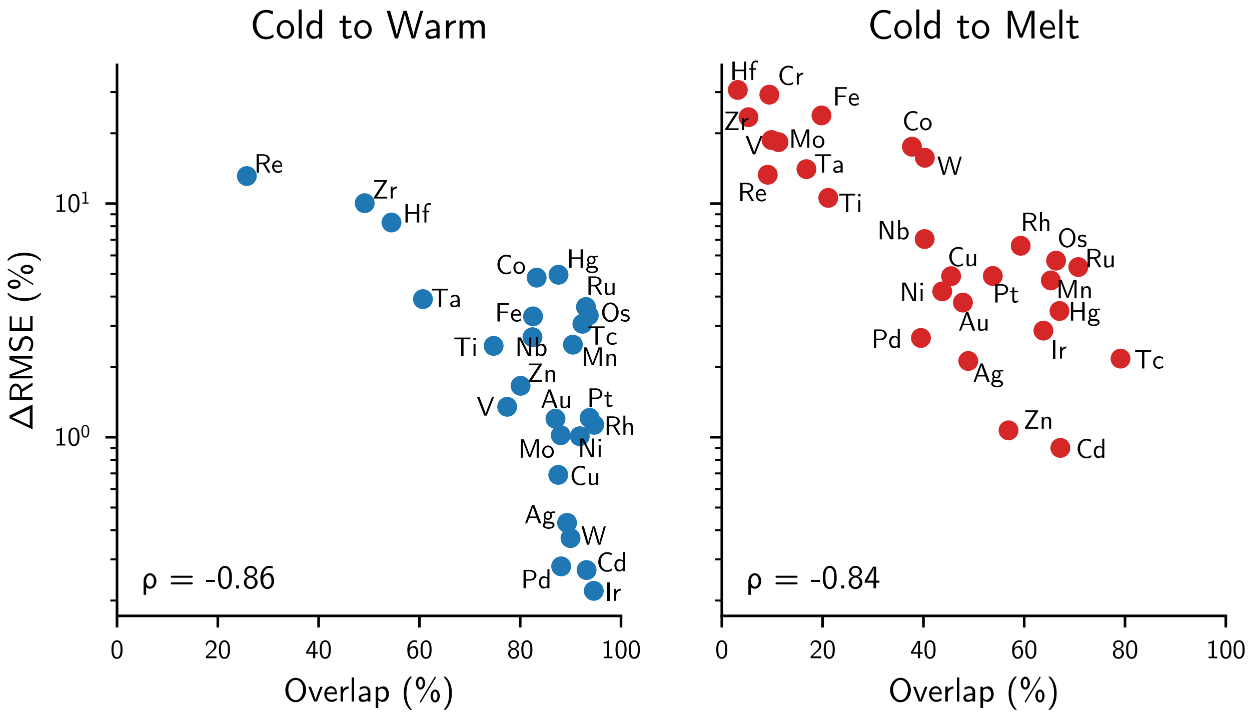

Fig. S24: Per-element overlap between train and test sets in the transferability experiments of TM23, and impact in the increase of relative root-mean-squared error (ΔRMSE). The left chart shows the per-element results for models tested on the "warm" subset, and the right chart shows the per-element results for models tested on the "melt" subset. Errors are obtained as reported by Owen *et al.*[25] The correlation coefficients are similar for both systems, suggesting they follow reasonably similar power laws. This figure is identical to Fig. 4f in the main text, but with labels.

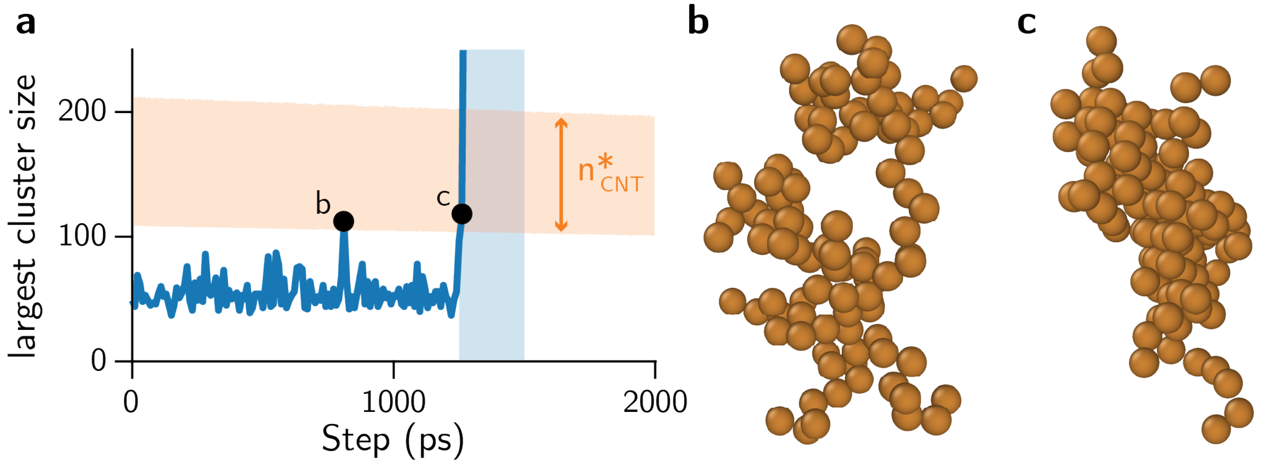

Fig. S25: **a**, maximum cluster size throughout the solidification trajectory, as depicted in Fig. 5f of the main paper. The black dots indicate two frames when the maximum cluster size surpasses the minimum required for nucleation n^*_{CNT} as per the classical nucleation theory (CNT). The visualization of these two clusters is shown in **b** and **c**. Whereas both have approximately the same number of atoms, **b** is much less compact compared to **c**, and may be better represented by two separate clusters instead of one. This may be an artifact of the graph-theoretical approach used to identify connected atoms in the simulation cell given values of the differential entropy $\delta\mathcal{H}$.

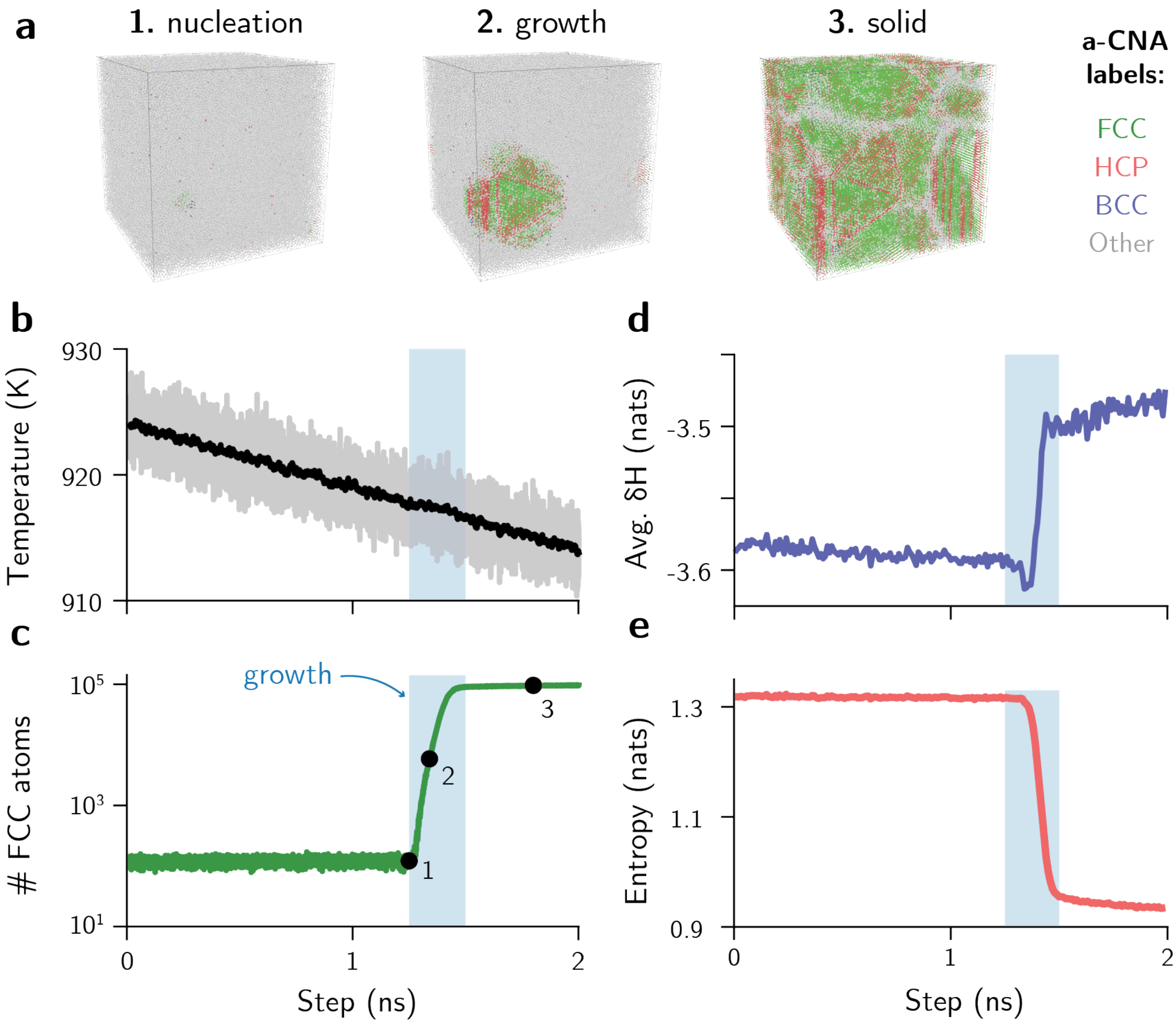

Fig. S26: **a**, Visualization of the solidification trajectory during the nucleation, growth, and solid states. Face-centered cubic (FCC), hexagonal close-packed (HCP), and body-centered cubic (BCC) phases are shown with green, red, and blue colors, respectively. Phases not identified by the adaptive common neighbor analysis (a-CNA)[27] are represented in gray. **b**, Average (black) and instantaneous (gray) temperature and **c**, number of FCC atoms derived from the molecular dynamics (MD) simulation. The shaded blue area indicates the time window where crystal growth is observed. The critical nucleus is observed around 917 K. The black dots indicate the frames corresponding to nucleation, growth, and final solidified system visualized in **a**. **d**, Average differential entropy $\delta\mathcal{H}$ using the first frame (melt) as reference for the entire solidification trajectory. The drop in the average $\delta\mathcal{H}$ around 1.25 ns suggests that the phases during growth are well-represented in the melt. **e**, Entropy computed for each frame using our information theoretical method.

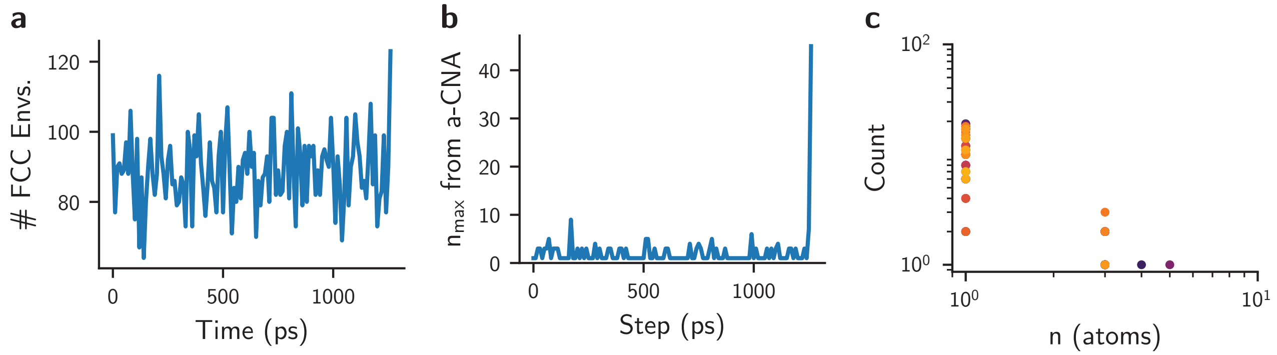

Fig. S27: **a**, Total number of face-centered cubic (FCC) environments in the Cu nucleation trajectory up until the nucleation event, as detected by the adaptive common neighbor analysis (a-CNA)[27] algorithm. The a-CNA algorithm predicts that less than 120 FCC-like environments are present in the simulation box, whereas the expected critical nucleus (i.e., values derived from the classical nucleation theory based on simulated and experimental parameters) should contain more than 100 atoms. **b**, Number of atoms n of the largest FCC cluster detected with a-CNA. Until nucleation, the cluster size remains at values close to 1, suggesting the absence of clusters. **c**, The results above lead to a cluster size distribution that barely has a distribution per se, as most frames contain only single-atom “FCC-like” environment. This figure indicates the cluster size distribution for multiple time steps (brighter colors indicate later frames, reproducing Fig. S28 below).

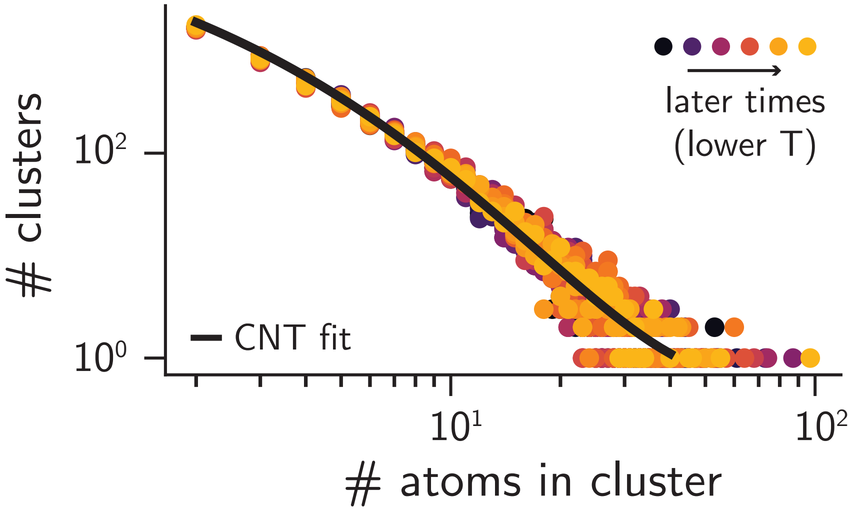

Fig. S28: Cluster size distribution in the melt prior to nucleation. The number of clusters (# clusters) depends on the number of atoms in each cluster (# atoms) according to a power law, similar to predictions from the classical nucleation theory (CNT, fitted black line).

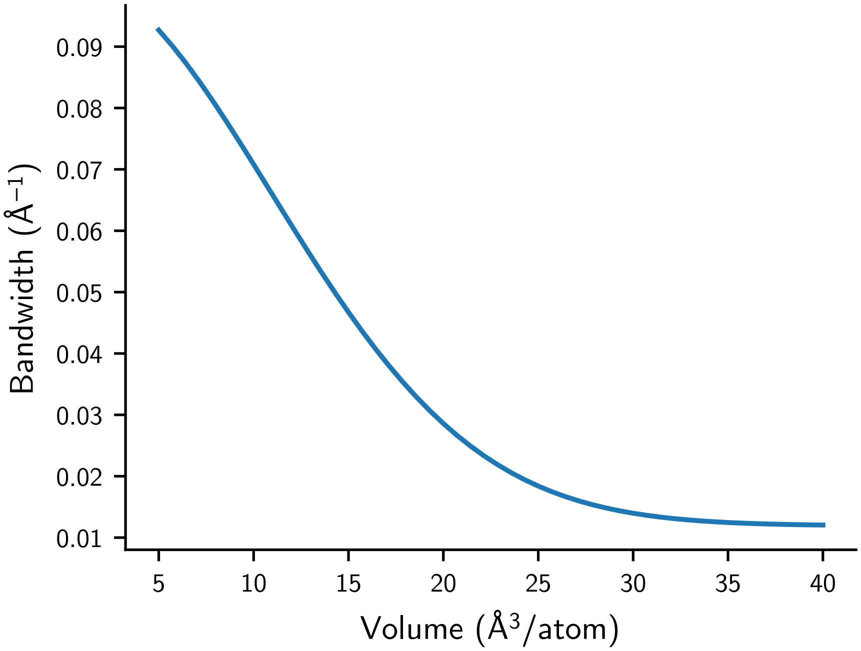

Fig. S29: Proposed dependence of the kernel bandwidth with the volume of the system for estimating the vibrational component of thermodynamic entropies from descriptors distributions. The bandwidth saturates at high volumes to ensure that residual information is captured from the data despite the non-thermodynamic behavior.

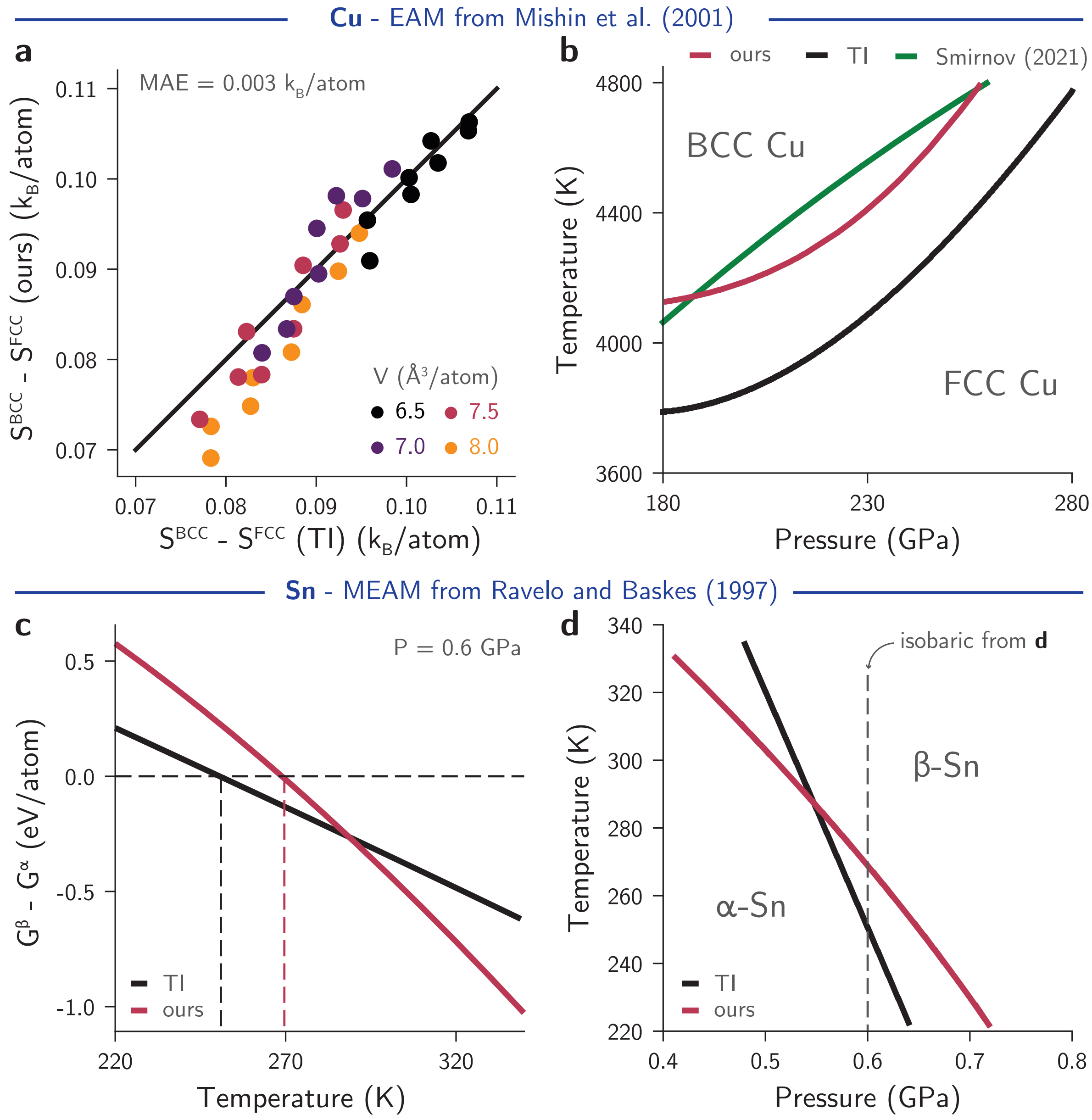

Fig. S30: **a**, Entropy differences between body-centered cubic (BCC) and face-centered cubic (FCC) copper at different temperatures and densities, as obtained by thermodynamic integration (TI) and our method, are nearly identical. Simulations of copper were performed using the embedded atom method (EAM) potential from Mishin et al.[10] Higher atomic volumes are shown with brighter colors. Different points with the same color correspond to different temperatures at the same volume. **b**, Phase boundaries of Cu computed using our method (red) and from TI (black) using a force field are similar in shape and ranges. A reference phase boundary from the literature, computed using density functional theory (DFT) and a quasiharmonic approximation from Smirnov,[11] is shown in green. **c**, Differences in Gibbs free energy (G) between α and β phases of Sn at a pressure (P) of 0.6 GPa using our method (red) and TI (black). Atomistic simulations of the solid Sn phases were performed with a modified embedded atom method (MEAM) for Sn from Ravelo and Baskes.[12] Despite the different approaches to compute G, the results are consistent in values and correctly predict a phase transformation around the same temperature ranges. **d**, The phase boundaries between α-Sn and β-Sn computed using our method (red) and TI (black) show good agreement across a range of pressures and temperatures.

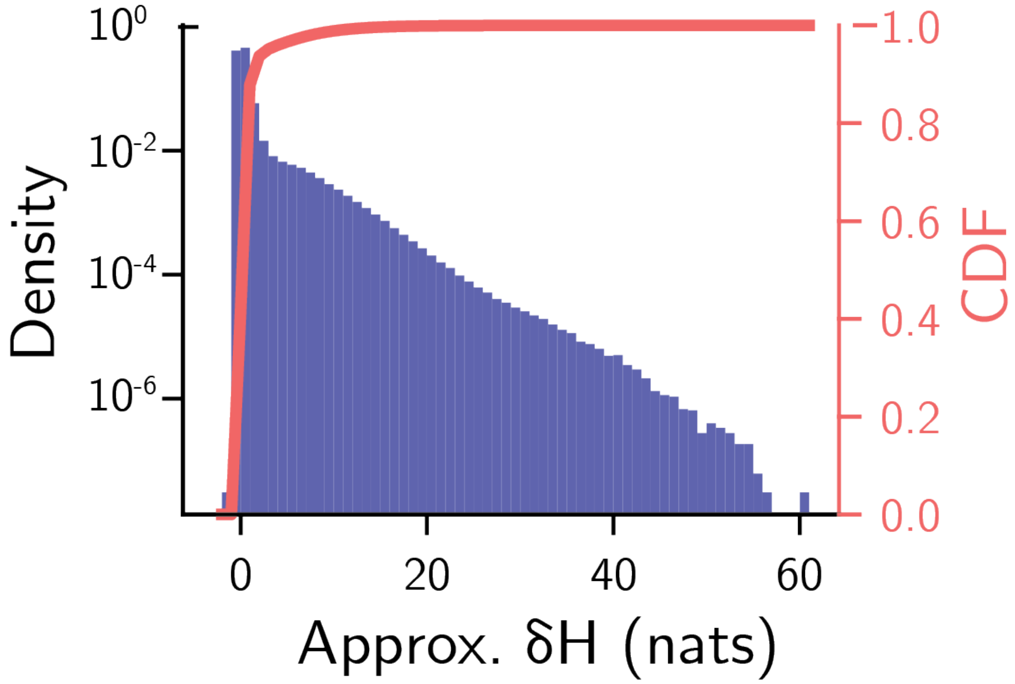

Fig. S31: Distribution (blue) and cumulative density function (CDF, red) of approximated values for the differential entropy $\delta\mathcal{H}$. 87% of the atoms exhibit $\delta\mathcal{H} < 0$ nats and thus are reasonably close to the training set.

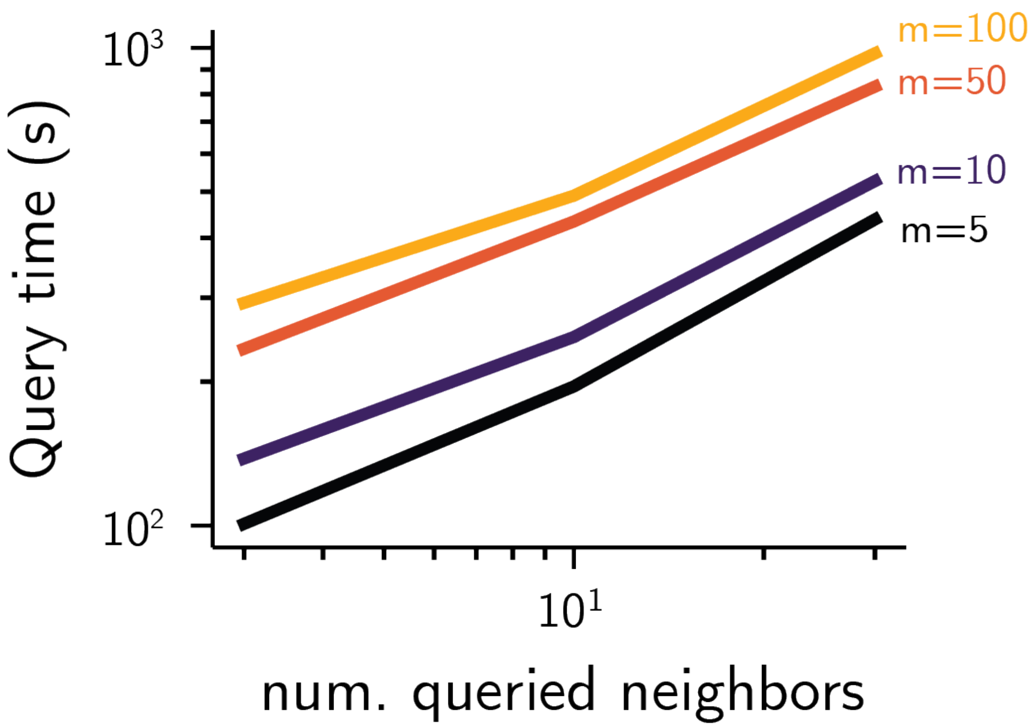

Fig. S32: Computational performance of the approximate nearest neighbors search. At the low-resource side ($N = 3$ queried neighbors per environment, index constructed with $m = 5$ neighbors), the values of differential entropy $\delta\mathcal{H}$ for all 32.5M atoms are evaluated in about 100 seconds when performed in a single node with 56 threads. For the Spectral Neighbor Analysis Potential (SNAP) dataset, the true $\delta\mathcal{H}$ for all environments is computed in about 255 s (wall-time) with the same hardware and parallelization settings.

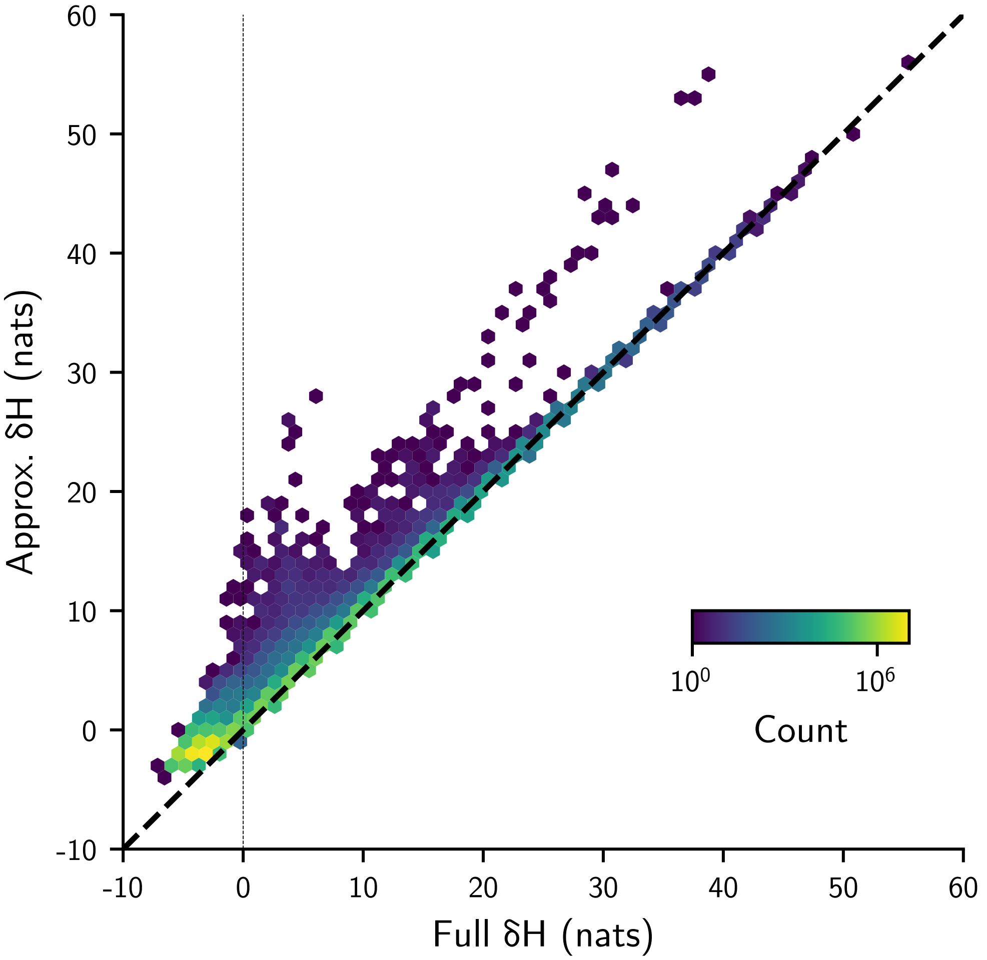

Fig. S33: Comparison between approximated value of differential entropy $\delta\mathcal{H}$ computed with a number of neighbors of $n = 30$ queried per data point and with $m = 50$ neighbors when constructing the index. The comparison shows that, in most cases, the use of approximate $\delta\mathcal{H}$ is a reasonable approximation. Importantly, due to the results in Eq. (S20), the approximation always overestimates the value of $\delta\mathcal{H}$, and thus can serve as an upper bound for the differential entropy.

D. Supplementary Tables

Table S1: Overlap between test and train sets for the TM23 dataset,[25] as computed by the fraction of differential entropy values larger than 0 ($\delta\mathcal{H} > 0$). The overlaps are provided in percentages (%).

Element	Full Dataset	Cold → Warm	Cold → Melt
Ag	99.6	89.3	48.9
Au	98.4	87.0	47.8
Cd	98.0	93.2	67.2
Co	97.0	83.3	37.7
Cr	95.4	87.6	9.4
Cu	99.5	87.5	45.5
Fe	95.8	82.5	19.8
Hf	94.8	54.5	3.2
Hg	97.3	87.6	67.0
Ir	99.8	94.6	63.8
Mn	98.9	90.5	65.3
Mo	96.6	88.0	11.2
Nb	95.3	82.4	40.2
Ni	99.4	91.8	43.7
Os	98.5	93.6	66.3
Pd	99.3	88.1	39.5
Pt	99.5	93.8	53.8
Re	97.9	25.8	9.1
Rh	99.5	94.7	59.3
Ru	98.6	93.0	70.7
Ta	96.0	60.7	16.8
Tc	98.5	92.3	79.1
Ti	95.1	74.7	21.1
V	94.8	77.4	9.9
W	99.0	90.0	40.3
Zn	97.2	80.1	56.9
Zr	93.5	49.1	5.3

References

[1] Widdowson, D. & Kurlin, V. Resolving the data ambiguity for periodic crystals. *Advances in Neural Information Processing Systems (NeurIPS 2022)* **35**, 24625–24638 (2022).

[2] Schwalbe-Koda, D., Widdowson, D. E., Pham, T. A. & Kurlin, V. A. Inorganic synthesis-structure maps in zeolites with machine learning and crystallographic distances. *Digital Discovery* **2**, 1911–1924 (2023). URL http://dx.doi.org/10.1039/D3DD00134B.

[3] Karabin, M. & Perez, D. An entropy-maximization approach to automated training set generation for interatomic potentials. *The Journal of Chemical Physics* **153** (2020).

[4] de Oca Zapiain, D. M. *et al.* Training data selection for accuracy and transferability of interatomic potentials. *npj Computational Materials* **8** (2022). URL https://doi.org/10.1038/s41524-022-00872-x.

[5] Beirlant, J., Dudewicz, E. J., Györfi, L., Van der Meulen, E. C. *et al.* Nonparametric entropy estimation: An overview. *International Journal of Mathematical and Statistical Sciences* **6**, 17–39 (1997).

[6] Oganov, A. R. & Valle, M. How to quantify energy landscapes of solids. *The Journal of Chemical Physics* **130**, 104504 (2009). URL https://doi.org/10.1063/1.3079326.

[7] Hsu, T. *et al.* Score-based denoising for atomic structure identification. *npj Computational Materials* **10**, 155 (2024).

[8] Dammak, H., Chalopin, Y., Laroche, M., Hayoun, M. & Greffet, J.-J. Quantum thermal bath for molecular dynamics simulation. *Physical Review Letters* **103**, 190601 (2009).

[9] Lindemann, F. A. Über die Berechnung molekularer Eigenfrequenzen. *Physikalische Zeitschrift* **11**, 609 (1910).

[10] Mishin, Y., Mehl, M., Papaconstantopoulos, D., Voter, A. & Kress, J. Structural stability and lattice defects in copper: Ab initio, tight-binding, and embedded-atom calculations. *Physical Review B* **63**, 224106 (2001).

[11] Smirnov, N. A. Relative stability of Cu, Ag, and Pt at high pressures and temperatures from ab initio calculations. *Physical Review B* **103**, 064107 (2021). URL https://link.aps.org/doi/10.1103/PhysRevB.103.064107.

[12] Ravelo, R. & Baskes, M. Equilibrium and Thermodynamic Properties of Grey, White, and Liquid Tin. *Physical Review Letters* **79**, 2482–2485 (1997). URL https://link.aps.org/doi/10.1103/PhysRevLett.79.2482.

[13] Thompson, A. P. *et al.* LAMMPS - a flexible simulation tool for particle-based materials modeling at the atomic, meso, and continuum scales. *Comp. Phys. Comm.* **271**, 108171 (2022).

[14] Freitas, R., Asta, M. & De Koning, M. Nonequilibrium free-energy calculation of solids using lammps. *Computational Materials Science* **112**, 333–341 (2016).

[15] Piessens, R., de Doncker-Kapenga, E., Überhuber, C. W. & Kahaner, D. K. *QUADPACK: a subroutine package for automatic integration*, vol. 1 (Springer Science & Business Media, 2012).

[16] Virtanen, P. *et al.* SciPy 1.0: Fundamental Algorithms for Scientific Computing in Python. *Nature Methods* **17**, 261–272 (2020).

[17] Pedregosa, F. *et al.* Scikit-learn: Machine learning in Python. *Journal of Machine Learning Research* **12**, 2825–2830 (2011).

[18] Schneider, T. & Stoll, E. Molecular-dynamics study of a three-dimensional one-component model for distortive phase transitions. *Physical Review B* **17**, 1302 (1978).

[19] Koenker, R. *Quantile Regression*. Econometric Society Monographs (Cambridge University Press, 2005).

[20] Dong, W., Moses, C. & Li, K. Efficient k-nearest neighbor graph construction for generic similarity measures. In *Proceedings of the 20th International Conference on World Wide Web*, 577–586 (2011).

[21] Chmiela, S. *et al.* Machine learning of accurate energy-conserving molecular force fields. *Science Advances* **3** (2017). URL https://www.science.org/doi/10.1126/sciadv.1603015.

[22] Christensen, A. S. & von Lilienfeld, O. A. On the role of gradients for machine learning of molecular energies and forces. *Machine Learning: Science and Technology* **1**, 045018 (2020). URL https://iopscience.iop.org/article/10.1088/2632-2153/abba6f.

[23] Batatia, I., Kovacs, D. P., Simm, G., Ortner, C. & Csanyi, G. MACE: Higher Order Equivariant Message Passing Neural Networks for Fast and Accurate Force Fields. In Koyejo, S. *et al.* (eds.) *Advances in Neural Information Processing Systems*, vol. 35, 11423–11436 (Curran Associates, Inc., 2022). URL https://proceedings.neurips.cc/paper_files/paper/2022/file/4a36c3c51af11ed9f34615b81edb5bbc-Paper-Conference.pdf.

[24] Rowe, P., Deringer, V. L., Gasparotto, P., Csányi, G. & Michaelides, A. An accurate and transferable machine learning potential for carbon. *The Journal of Chemical Physics* **153** (2020).

[25] Owen, C. J. *et al.* Complexity of many-body interactions in transition metals via machine-learned force fields from the TM23 data set. *npj Computational Materials* **10**, 92 (2024).

[26] Batzner, S. *et al.* E(3)-equivariant graph neural networks for data-efficient and accurate interatomic potentials. *Nature Communications* **13**, 2453 (2022). URL https://www.nature.com/articles/s41467-022-29939-5.

[27] Stukowski, A. Structure identification methods for atomistic simulations of crystalline materials. *Modelling and Simulation in Materials Science and Engineering* **20**, 045021 (2012).
